# Supplementary material for: Impact of the COVID-19 pandemic on intimate partner violence during pregnancy: evidence from a multimethods study of recently pregnant women in Ethiopia
Source: BMJ Open. 2022 Apr 11;12(4):e055790. doi: 10.1136/bmjopen-2021-055790 (PMC9006189; doi:10.1136/bmjopen-2021-055790)
Supplement: Author's manuscript [file bmjopen-2021-055790.draft_revisions.pdf]

BMJ Open is committed to open peer review. As part of this commitment we make the peer review history of every article we publish publicly available.

When an article is published we post the peer reviewers' comments and the authors' responses online. We also post the versions of the paper that were used during peer review. These are the versions that the peer review comments apply to.

The versions of the paper that follow are the versions that were submitted during the peer review process. They are not the versions of record or the final published versions. They should not be cited or distributed as the published version of this manuscript.

BMJ Open is an open access journal and the full, final, typeset and author-corrected version of record of the manuscript is available on our site with no access controls, subscription charges or pay-per-view fees (http://bmjopen.bmj.com).

If you have any questions on BMJ Open's open peer review process please email info.bmjopen@bmj.com

BMJ Open

Impact of COVID-19 on intimate partner violence during pregnancy: evidence from a mixed-methods study of recently pregnant women in Ethiopia

Journal:	*BMJ Open*
Manuscript ID	bmjopen-2021-055790
Article Type:	Original research
Date Submitted by the Author:	27-Jul-2021
Complete List of Authors:	Wood, Shannon; Johns Hopkins University Bloomberg School of Public Health<br>Yirgu, Robel; Addis Ababa University<br>Wondimagegnehu, Abigiya; Addis Ababa University<br>Qian, Jiage; Johns Hopkins University Bloomberg School of Public Health<br>Milkovich, Rachel; Johns Hopkins University Bloomberg School of Public Health<br>Decker, Michele; Johns Hopkins University Bloomberg School of Public Health<br>Glass, Nancy; Johns Hopkins University School of Nursing<br>Seid, Fatuma; Ethiopia Ministry of Health<br>Zekarias, Lensa; Ethiopia Ministry of Health<br>Zimmerman, Linnea; Johns Hopkins University Bloomberg School of Public Health, Population Family and Reproductive Health
Keywords:	COVID-19, Public health < INFECTIOUS DISEASES, QUALITATIVE RESEARCH, PUBLIC HEALTH, REPRODUCTIVE MEDICINE, SEXUAL MEDICINE

SCHOLARONE™
Manuscripts

I, the Submitting Author has the right to grant and does grant on behalf of all authors of the Work (as defined in the below author licence), an exclusive licence and/or a non-exclusive licence for contributions from authors who are: i) UK Crown employees; ii) where BMJ has agreed a CC-BY licence shall apply, and/or iii) in accordance with the terms applicable for US Federal Government officers or employees acting as part of their official duties; on a worldwide, perpetual, irrevocable, royalty-free basis to BMJ Publishing Group Ltd ("BMJ") its licensees and where the relevant Journal is co-owned by BMJ to the co-owners of the Journal, to publish the Work in this journal and any other BMJ products and to exploit all rights, as set out in our licence.

The Submitting Author accepts and understands that any supply made under these terms is made by BMJ to the Submitting Author unless you are acting as an employee on behalf of your employer or a postgraduate student of an affiliated institution which is paying any applicable article publishing charge ("APC") for Open Access articles. Where the Submitting Author wishes to make the Work available on an Open Access basis (and intends to pay the relevant APC), the terms of reuse of such Open Access shall be governed by a Creative Commons licence – details of these licences and which Creative Commons licence will apply to this Work are set out in our licence referred to above.

Other than as permitted in any relevant BMJ Author's Self Archiving Policies, I confirm this Work has not been accepted for publication elsewhere, is not being considered for publication elsewhere and does not duplicate material already published. I confirm all authors consent to publication of this Work and authorise the granting of this licence.

For peer review only

Title: Impact of COVID-19 on intimate partner violence during pregnancy: evidence from a mixed-methods study of recently pregnant women in Ethiopia

Authorship team: Shannon N. Wood[1], Robel Yirgu[2], Abigiya Wondimagegnehu[2], Jiage Qian[1], Rachel Milkovich[1], Michele R. Decker[1,3,4], Nancy Glass[4,5], Fatuma Seid[6], Lensa Zekarias[6], Linnea A. Zimmerman[1]

Affiliations:
[1] Department of Population Family and Reproductive Health, Johns Hopkins Bloomberg School of Public Health, Baltimore, Maryland, USA.
[2] Addis Ababa University School of Public Health, Addis Ababa, Ethiopia.
[3] Center for Public Health and Human Rights, Johns Hopkins Bloomberg School of Public Health, Baltimore, Maryland, USA.
[4] Johns Hopkins School of Nursing, Baltimore, Maryland, USA.
[5] Center for Global Health, Johns Hopkins University, Baltimore, Maryland, USA.
[6] Ethiopia Federal Ministry of Health, Addis Ababa, Ethiopia.

Corresponding Author:
Shannon N. Wood
615 N. Wolfe Street
Baltimore, MD
swood@jhu.edu

Word count: 2778
Figures: 1
Tables: 2

For peer review only

For peer review only - http://bmjopen.bmj.com/site/about/guidelines.xhtml

Abstract

Objectives: This mixed-methods study aimed to: 1) compare the prevalence of intimate partner violence (IPV) during pregnancy pre- and during-COVID-19 using quantitative data; 2) contextualize pregnant women's IPV experiences during COVID-19 through supplemental interviews.

Design: Analyses uses Performance Monitoring for Action (PMA)-Ethiopia data, a cohort of 2,873 pregnant women that collects data at pregnancy, 6-weeks, 6-months, and 1-year postpartum. Following 6-week postpartum interview, in-depth interviews contextualized experiences of IPV during pregnancy with a subset of participants (n=24).

Participants: All pregnant women residing within six regions of Ethiopia, covering 91% of the population, were eligible for the cohort study (n=2,868 completed baseline survey). Quantitative analyses were restricted to the 2,388 women with complete six-week survey data (retention=82.7%). A purposive sampling frame was used to select qualitative participants on baseline survey data, with inclusion criteria specifying completion of quantitative 6-week post-COVID interview and indication of IPV experience.

Interventions: A State of Emergency in Ethiopia was declared in response to COVID-19 approximately halfway through 6-week postpartum interview, enabling a natural experiment (n=1,405 pre-COVID-19; n=983 during-COVID).

Primary outcome measures: IPV during pregnancy was assessed via the 10-item Revised Conflict and Tactics Scale (CTS-2).

Results: One in ten women experienced any IPV during pregnancy prior to COVID (10.5%), and prevalence of IPV during pregnancy increased to 15.1% during COVID (aOR=1.51; p=0.02). Stratified by residence, odds of IPV during COVID increased for urban women only (aOR=2.09; p=0.03), however, IPV prevalence was higher in rural regions at both timepoints. Qualitative data reveal COVID-related stressors namely, loss of household income and increased time spent within the household, exacerbated IPV.

Conclusions: These mixed-methods results highlight the prevalent, severe violence that pregnant Ethiopian women experience, with COVID-related increases concentrated in urban areas. Integration of IPV response and safety planning across the continuum of care can mitigate impact.

For peer review only

For peer review only - http://bmjopen.bmj.com/site/about/guidelines.xhtml

Article summary

Strengths and limitations of this study

- Natural experiment based on split of pre-existing cohort to examine differences in intimate partner violence (IPV) during pregnancy pre/post COVID-19 restrictions
- Large quantitative sample inclusive of all pregnant women residing within six regions of Ethiopia, covering 91% of the population
- In-depth interviews to contextualize women's experiences with IPV during pregnancy during COVID-19
- Groupings may not exactly estimate pre/post exposure and no women who were truly only exposed to IPV during COVID-19
- Qualitative interviews only occurred within two regions and are not generalizable to all pregnant women experiencing IPV during pregnancy

For peer review only

Introduction

Pandemics exacerbate gender inequities, including intimate partner violence (IPV) (1–3). Evidence from previous epidemics and regional crises indicates heightened IPV via increased economic insecurity, social isolation, and exposure to perpetrators, and more limited options for garnering support (1). COVID-19 is no exception—12 out of 15 early studies indicate increases in IPV globally (4).

The pregnancy and postpartum period are pivotal timepoints for assessing and responding to IPV, given health impacts to mother and baby, and multiple points of contact with healthcare providers. Global estimates indicate that 2-14% of women experience violence during pregnancy (5,6). Pre-existing IPV may be sustained or exacerbated, and abusive partners limit access to antenatal and postnatal care (6). IPV during pregnancy incurs profound effects, including miscarriage, premature labor, low birthweight, and maternal depression (7–10). Timely response is thus critical for this vulnerable sub-population (5,10). Linkage to woman-centered care may decrease subsequent abuse (10).

Violence prevention and response is critical within the Ethiopian context. Ethiopia Demographic and Health Survey (DHS) data indicate that 27% of ever-married women age 15-49 have experienced IPV within the past year and 4% experienced IPV during pregnancy (11). In April 2020, Ethiopia declared a State of Emergency in response to COVID-19 (12); though essential to limit infection, lockdown measures may exacerbate pregnant women's IPV experiences (2,3). Early evidence from Amhara, Ethiopia indicated similar levels of IPV pre- and during COVID (13), while 18% of antenatal care attendees in Addis Ababa reported perceived increase in violence during pregnancy since the onset of the pandemic (14). Both studies, however, were limited in generalizability due to cross-sectional design and sampling considerations.

While global evidence indicates increases in IPV, no studies aimed at understanding the impact of COVID-19 or related response measures examine violence during pregnancy nor have been conducted within the Ethiopian context (4). Ethical standards advocate for continued IPV monitoring using existing study infrastructures if the research aims to understand the magnitude of IPV burden and linkages with social/economic factors; informs response efforts; and meets ethical obligations (1,15). Utilizing an existing national cohort of recently pregnant women and rigorous ethical standards, this study aimed to examine how the COVID-19 pandemic affected pregnant women's experiences with IPV through 1) comparison of IPV prevalence pre- and post-COVID-19 using quantitative data and 2) contextualization of women's experiences of IPV during COVID-19 through exploration of lockdown measures.

Methods

Study Design

This analysis is situated within the Performance Monitoring for Action (PMA)-Ethiopia cohort study, a collaboration between Johns Hopkins Bloomberg School of Public Health (JHSPH), Addis Ababa University (AAU), and the Ethiopian Federal Ministry of Health (FMoH). PMA-Ethiopia collects data on a cohort of 2,879 pregnant women at pregnancy, 6-weeks, 6-months, and 1-year postpartum. Enrollment into the cohort began in October 2019. The full protocol for PMA Ethiopia is detailed elsewhere (16). This analysis utilizes quantitative 6-week postpartum data to allow for most comprehensive measurement of violence throughout the entire pregnancy period. Following 6-week interview, in-depth interviews contextualized experiences of IPV during pregnancy, among a subset of participants (n=24). Institutional Review Board approval was obtained at both JHSPH (IRB00013278) and AAU College of Health Sciences (077/20/SPH), and protocols were implemented in line with best practices for violence research (17).

Participants

All pregnant women residing within six regions of Ethiopia that covers 91% of the population were eligible for the cohort study (n=2,868 completed baseline survey). Quantitative analyses were restricted to the 2,388 women with complete six-week survey data (retention=82.7%). A State of Emergency in Ethiopia was

For peer review only - http://bmjopen.bmj.com/site/about/guidelines.xhtml

declared in response to COVID-19 approximately halfway through fielding the 6-week postpartum interview (April 8, 2020), thus enabling a natural experiment. Specifically, 1,405 6-week interviews were conducted pre-COVID-19; the remaining 983 occurred after COVID-19 emergency lockdown procedures eased in early June.

Measures

Intimate partner violence, the primary outcome of interest, was measured via the 10-item Revised Conflict and Tactics Scale (18), which asks about specific violence behaviors at any time during pregnancy, per best practices for violence research (17).Three violence measures were examined: 1) any IPV, 2) any physical IPV, 3) any sexual IPV; affirmative response to any behavior was classified as IPV experience. Physical and sexual IPV were derived from the following items: 1) physical IPV: "push you, shake you, or throw something at you;" "slap you;" "twist your arm or pull your hair;" "punch you with his fist or with something that could hurt you;" "kick you, drag you, or beat you up;" "try to choke you or burn you on purpose;" "threaten or attack you with a knife, gun, or other weapon" and 2) sexual IPV: "physically force you to have sexual intercourse with him when you did not want to;" "physically force you to perform any other sexual acts you did not want to;" "used threats or pressure to make you have sex when you did not want to." Any physical and any sexual IPV were not mutually exclusive.

The primary exposure variable of interest measures pre/post exposure to COVID-19, a binary variable, indicating whether a woman completed her 6-week postpartum survey before (pre-lockdown) or after (post-lockdown) April 8, when the State of Emergency was declared and data collection paused.

Analyses were stratified by urban/rural residence given the differences in COVID-19 lockdown measures. Sociodemographic variables explored as adjustment variables were chosen on a conceptual basis, and included region, household wealth, age, parity, and education; all were examined in categorical form.

Statistical Analysis

Descriptive statistics examined the distribution of sociodemographic characteristics by pre/post exposure; design-based F statistics assessed whether distributions were similar. Venn diagrams classified violence experiences during each timepoint. Next, bivariate distributions of each type of violence were examined by exposure, overall and stratified by urban/rural residence. Bivariate and multivariable logistic regression models were used to examine differences in violence experience pre/during COVID and residence; for adjusted models, correlations between covariates were examined for multi-collinearity, with only residence and . All analyses were conducted in STATA 16, with statistical significance set at $p<0.05$, and accounting for complex-survey design.

Qualitative Data Collection and Analysis

To contextualize the impact of COVID-19 on women's experiences of violence during pregnancy, an explanatory qualitative phase was conducted. Specifically, 24 semi-structured qualitative interviews focused on IPV were conducted in the month following 6-week quantitative data collection in Oromiya and SNNP regions; regions were selected based on high IPV during pregnancy from baseline survey and feasibility. At the 6-week survey, participants were consented for potential follow-up specific to partner-related items. A purposive sampling frame was used to select participants on baseline survey data, with inclusion criteria specifying: 1) completion of quantitative 6-week post-COVID interview; 2) indication of IPV experience via quantitative data. Data collection continued until feasible sample size was met (n=14 Oromiya; n=10 SNNP) (19).

Training for the qualitative phase preceded data collection with focus on probing, ethical principles for IPV research, and research team protections. Semi-structured interview guides focused on women's experiences with IPV and IPV services. Participants were called prior to interview for scheduling considerations. All

For peer review only - http://bmjopen.bmj.com/site/about/guidelines.xhtml

interviews lasted approximately 25-30 minutes. Four trained interviewers used a structured note-taking tool to allow probing of experiences, while permitting rapid analysis for timely results (20). Immediately post-interview, interviewers typed and translated field notes.

Two researchers trained in qualitative analysis coded 24 structured notes using Atlas.ti software. Inductive thematic analysis was used to identify emergent themes and sub-themes and to create an initial set of codes. Dual coding and retro-coding were used to enhance agreement between coders. Coders also met and collaborated regularly with the field research team to discuss and clarify interpretation. Coding was complete when saturation of themes was achieved (21); illustrative quotes were then downloaded from Atlas.ti and organized in matrices of code themes that were organized by IPV experience.

Patient and Public Involvement

Community members are not directly involved in PMA-Ethiopia research, however, a Project Advisory Board, including members of the Federal Ministry of Health, health providers, and key stakeholders, are consulted about country-specific priorities, including IPV, during survey development. Results are disseminated to the advisory board to inform action.

Results

Demographic characteristics of study participants by pre/post exposure are presented in Table 1. Significant differences were observed for parity only, where women of higher parity were more likely to be interviewed pre-COVID.

Figure 1 displays overlap of types of violence experienced by women during pregnancy, by exposure. At both timepoints, sexual IPV ($7.4\%_{\text{pre-COVID}}$; $9.8\%_{\text{during-COVID}}$) was higher than physical IPV ($5.0\%_{\text{pre-COVID}}$; $7.8\%_{\text{during-COVID}}$) and often occurred in isolation.

[insert Figure 1 here]

Bivariate and logistic regression results comparing pre-COVID IPV to during COVID IPV, overall and by residence, are presented in Table 2. One in ten women experienced any IPV during pregnancy prior to COVID (10.5%), while this increased to 15.1% during COVID-19 (aOR=1.51; 95% CI=1.06-2.15; p=0.02). When stratified by residence, odds of IPV during COVID, compared to pre-COVID, increased for urban women (aOR=2.09; 95%CI=1.10-3.96; p=0.03), while odds slightly attenuated for rural women, but the difference did not achieve statistical significance (aOR=1.43; 95% CI=0.96-2.13; p=0.08). IPV prevalence was consistently higher among rural women.

In examining violence sub-types, physical IPV significantly increased overall (aOR=1.60; 95% CI=1.07-2.40; p=0.02) and in urban areas (aOR=2.46; 95% CI=1.18-5.10; p=0.02). Significant changes were not observed for physical violence in rural areas or for sexual violence.

Qualitative data indicate that COVID-19 affected IPV within relationships differentially based on husband's job status. Women whose husbands experienced loss of income or employment due to the pandemic often reported that COVID-19 put stress on their relationship and increased instances of physical violence.

> Since he was not working, he didn't have money; it was difficult for us to pay the house rent, it was difficult to buy food. These problems created conflict, then he insulted and hit me.

For peer review only - http://bmjopen.bmj.com/site/about/guidelines.xhtml

Many women experienced an increase in verbal abuse due to COVID-related stressors. Several participants shared that the main reason for conflict prior to the pandemic were disagreements about household responsibilities, suggesting that COVID-19 exacerbated this tension.

> The violence increased because we stayed together at our house because there was movement restriction in our village so he couldn't go to the market to sell goats and sheeps. So, our income decreased a lot and because of that we had arguments most of the time.

The pandemic also changed the amount of time that spouses spent at home together, most often due to a husband's loss of employment. One participant disclosed that this gave her husband an opportunity to further control her activities and to engage in sexual violence at a high frequency.

> During corona time, he stays at home so he is happy with that because he can control all my day-to-day activities. No physical and psychological violence happened but sometimes there is sexual violence. It might be four times a week.

It should be noted that several participants stated that the pandemic had little or no impact on their relationships with their husbands, however, previous violence persisted throughout.

> We had a disagreement during this pregnancy. It was during coronavirus. But the cause was not related with corona. We disagreed due to children. The corona pandemic has nothing to do with our relationship.

In many instances, the violence described by women during pregnancy was severe.

> When I became pregnant, the part of my body he hits was changed but not the frequency. He used to beat every part of my body but when I became pregnant, he especially didn't hit me around the belly. Because he cares for the baby. He slaps my face with my own hand.

> He doesn't care if I am pregnant or not. When he is very angry, he gets very emotional and throws at me whatever he finds, whether it is a chair or anything. I had stillbirth, the fetus died in the belly, though I had a spontaneous vaginal delivery. When he heard the baby died in my uterus, he got angry and thought I was the one who killed the baby.

> When I was nine months pregnant; he hits me and throw me on the stone then I went to my father's home. Immediately, my father's neighbors took me to hospital. I was admitted there for 15 days.

Discussion

These mixed-methods results highlight the prevalent, severe violence that women experience at a critical, and often vulnerable, period during their reproductive lives. Utilizing a natural experiment embedded in cohort of recently pregnant women in the six largest regions of Ethiopia, we found heightened IPV during COVID, compared to the pre-COVID period, with greatest between-time impact concentrated in urban areas. Discrepancies by residence corroborate heightened cases and more COVID-19 preventive measures within urban areas (12).Qualitative data reveal the mechanisms through COVID-related stressors operate to exacerbate IPV, namely loss of household income and increased time spent within the household.

More than one in ten women experienced violence during pregnancy within Ethiopia ($10.5\%_{\text{pre-COVID}}$)—IPV during pregnancy reported by recently pregnant women in this study was substantially higher than 2016 DHS lifetime estimates (3.7%) (11). Continuous monitoring within national surveillance systems and large-scale cohort studies can assist identifying who is at risk for IPV during most recent pregnancy.

For peer review only - http://bmjopen.bmj.com/site/about/guidelines.xhtml

Utilizing best practices for violence-related research (15,17), e.g. timely linkage to woman-centered referrals, can bolster safety and minimize adverse outcomes.

Given distinct patterns of IPV by urban and rural residence, interventions must be tailored by locality and women's circumstances. The impact of COVID-19 on violence during pregnancy was concentrated primarily within urban settings. Immediate intervention and connection to IPV referral services should focus on urban settings, prone to closures and economic impact. As time and household finances were indicated as stressors, temporary separation and seeking support from family members and neighbors may be a feasible strategy to minimize impact of violence—this safety strategy has been useful for women experiencing IPV in urban informal settlements of Nairobi and other low- and middle-income contexts where leaving the relationship is not feasible or socially acceptable (22,23). Notably, however, IPV during pregnancy was highest for rural women at both timepoints (16.2%$_{rural}$ vs. 10.9%$_{urban}$ during COVID-19). Heightened prevalence within rural settings likely speaks to cohesive, patriarchal community norms promoting violence against women (24); in order to ultimately reduce IPV, large-scale transformative community norms interventions, such as Communities Care or SASA! (25,26), will be required.

This study is not without limitations—namely, these groupings may not exactly estimate pre/post exposure and we have no women who were truly only exposed to IPV during COVID-19. Further, our sample size was limited for additional subgroup analysis to examine women most at risk for IPV during pregnancy by pre-COVID and during-COVID time points. To maximize women's confidentiality and bolster safety in line with best practices for violence-related research (17), interviewers were instructed to not conduct either quantitative or qualitative interviews within the presence of a partner; given COVID lockdown measures and potential for controlling behaviors within abusive partnerships, our results may be an underestimate. Lastly, qualitative data collection occurred only within two of the six regions based on baseline prevalence of IPV during pregnancy—this purposive sampling strategy may have excluded women needing IPV support during COVID-19 and is not generalizable to all pregnant women experiencing IPV in Ethiopia.

While IPV during pregnancy increased overall during COVID-19, violence and related gender-based power disparities were prominent prior to the onset of the pandemic—accordingly, IPV prevention and response efforts in Ethiopia must be sustained in the post-COVID-19 era. We offer two concrete recommendations for maternal health providers. First, while antenatal care is a critical intervention point for identifying and providing care for women experiencing IPV during pregnancy, providers across the maternal and neonatal continuum of care must similarly provide support. Integration of IPV screening and psychological care into postnatal care is critical given links to postpartum depression (27,28). Clinic-based aids, including World Health Organization recommendations for clinical IPV identification and linkage to health and economic referrals, can be valuable in training providers to support survivors (29). Second, the use of safety decision-aids by providers across the continuum of care could assist in helping women assess their circumstances and level of danger, and create safety plans tailored to their situations. Safety planning with trained community health workers was found to increase safety preparedness in Kenya (30), and may be similarly valuable in rural areas of Ethiopia with trained Health Extension Workers. Clinic-based interventions to reduce and address IPV should not occur separately from other health services, but as part of larger community-led behavioral change programs with influential community members on targeting harmful gender norms and IPV, in order to ultimately empower women and girls. The COVID-19 pandemic has alerted the global community to pervasiveness of violence against women—continued momentum and investment is needed to mitigate its harmful impact.

For peer review only - http://bmjopen.bmj.com/site/about/guidelines.xhtml

Contributorship Statement: SNW designed the study, oversaw study details, and drafted the manuscript. RY oversaw all aspects of qualitative training and data collection. AW conducted training and data collection. JQ and RM conducted qualitative coding. MRD and NG served as ethical supervisors in the project. FS and LZ provided technical insight into Ethiopian IPV response systems. LAZ served as the Principal Investigator for the parent study and provided technical assistance. All authors participated in writing and approving the final manuscript.

Competing Interests: None declared.

Funding: Bill and Melinda Gates Foundation [INV 009466]and the Johns Hopkins Specialized Center for Research Excellence in Sex Differences (U54AG062333). Under the grant conditions of the Bill and Melinda Gates Foundation, a Creative Commons Attribution 4.0 Generic License has already been assigned to the Author Accepted Manuscript version that might arise from this submission.

Data Sharing Agreement: Quantitative data are available upon request from pmadata.org. Qualitative data are not available to maximize participant confidentiality.

Ethics Approval Statement: Johns Hopkins Bloomberg School of Public Health (IRB00013278) and Addis Ababa University College of Health Sciences (077/20/SPH)

For peer review only

References

1. Peterman A, Potts A, Donnell MO, Shah N, Oertelt-prigione S, Gelder N Van. Working Paper 528: Pandemics and Violence Against Women and Children. Washington, DC;
2. Gelder N Van, Peterman A, Potts A, Donnell MO, Thompson K, Shah N, et al. COVID-19: Reducing the risk of infection might increase the risk of intimate partner violence. EClinicalMedicine. 2020;21.
3. Roesch E, Amin A, Gupta J, Garcia-Moreno C. Violence against women during covid-19 pandemic restrictions. BMJ. 2020;369(m1712):1–2.
4. Bourgault S, Peterman A, O'Donnell M. Violence Against Women and Children During COVID-19 — One Year On and 100 Papers In: A Fourth Research Round Up. 2021.
5. Devries KM, Kishor S, Johnson H, Stöckl H, Bacchus LJ, Garcia-Moreno C, et al. Intimate partner violence during pregnancy: analysis of prevalence data from 19 countries. Reprod Health Matters. 2010;18(36):158–70.
6. García-Moreno C, Jansen HA, Ellsberg M, Heise L, Watts C. WHO multi-country study on women's health and domestic violence against women. Geneva; 2005.
7. Hill A, Pallitto C, Mccleary-Sills J, Garcia-moreno C. A systematic review and meta-analysis of intimate partner violence during pregnancy and selected birth outcomes. Int J Gynecol Obstet. 2016;133:269–76.
8. Alhusen JL, Ray E, Sharps P, Bullock L. Intimate Partner Violence During Pregnancy: Maternal and Neonatal Outcomes. J Women. 2015;24(1):100–6.
9. Berhanie E, Gebregziabher D, Berihu H, Gerezgiher A, Kidane G. Intimate partner violence during pregnancy and adverse birth outcomes : a case-control study. 2019;1–9.
10. Chisholm C, Bullock L, Ferguson II JE. Intimate partner violence and pregnancy: epidemiology and impact. Am J Obstet Gynecol. 2017;(August):141–4.
11. Central Statistical Agency and ICF. Ethiopia Demographic and Health Survey. Addis Ababa, Ethiopia and Rockville, Maryland, USA; 2016.
12. Ethiopia COVID19 Monitoring. 2020.
13. Tadesse AW, Tarekegn SM, Wagaw GB, Muluneh MD, Kassa AM. Prevalence and Associated Factors of Intimate Partner Violence Among Married Women During COVID-19 Pandemic Restrictions: A Community-Based Study. J Interpers Violence. 2020;
14. Teshome A, Gudu W, Bekele D, Asfaw M, Enyew R, Compton SD. Intimate partner violence among prenatal care attendees amidst the COVID-19 crisis: The incidence in Ethiopia. Int J Gynecol Obstet. 2021;153(1):45–50.
15. Peterman A, Bhatia A, Guedes A. Remote data collection on violence against women during COVID-19: A conversation with experts on ethics, measurement & research priorities.
16. Zimmerman L, Desta S, Yihdego M, Rogers A, Amogne A, Karp C, et al. Protocol for PMA-Ethiopia: A New Data Source for Cross-sectional and Longitudinal Data of Reproductive, Maternal, and Newborn Health. Accept Glob Heal Sci Pract. 2020;
17. World Health Organization (WHO). Ethical and safety recommendations for intervention research on violence against women. Geneva; 2016.
18. Straus M, Hamby S, Boney-McCoy S, Sugarman D. The Revised Conflict Tactics Scales (CTS2). J Fam Issues. 1996;17(3):283–316.
19. Guest G, Bunce A, Johnson L. How Many Interviews Are Enough? An Experiment with Data Saturation and Variability. Field methods. 2006;18(1):59–82.
20. Johnson BD, Dunlap E, Benoit E. Structured Qualitative Resesarch: Organizing "Mountains of Words" for Data Analysis, both Qualitative and Quantitative. Subst Use Misuse. 2010;45(5):648–70.
21. Guest G, MacQueen K, Namey E. Applied thematic analysis. Thousand Oaks: Sage Publications; 2012.
22. Wood SN, Kennedy RS, Hameeduddin Z, Asira B, Tallam C, Akumu I, et al. "Being married doesn't mean you have to reach the end of the world:" Safety planning with intimate partner

For peer review only - http://bmjopen.bmj.com/site/about/guidelines.xhtml

violence survivors and service providers in three urban informal settlements in Nairobi, Kenya. J Interpers Violence. 2019;Epub ahead of print.

23. Wood SN, Glass N, Decker MR. An integrative review of safety strategies for women experiencing intimate partner violence in low- and middle-income countries. Trauma, Violence, Abus. 2019;1–15.

24. Murphy M, Jones N, Yadete W, Baird S. Gender-norms, violence and adolescence: Exploring how gender norms are associated with experiences of childhood violence among young adolescents in Ethiopia. Glob Public Health. 2021;16(6):842–55.

25. Abramsky T, Devries K, Kiss L, Nakuti J, Kyegombe N, Starmann E, et al. Findings from the SASA ! Study : a cluster randomized controlled trial to assess the impact of a community mobilization intervention to prevent violence against women and reduce HIV risk in Kampala , Uganda. 2014;1–17.

26. Glass N, Perrin N, Clough A, Desgroppes A, Kaburu FN, Melton J, et al. Evaluating the communities care program: Best practice for rigorous research to evaluate gender based violence prevention and response programs in humanitarian settings. Confl Health. 2018;12(5):1–10.

27. Ashenafi W, Mengistie B, Egata G, Berhane Y. The role of intimate partner violence victimization during pregnancy on maternal postpartum depression in Eastern Ethiopia. SAGE Open Med. 2021;9:1–12.

28. Bitew T, Hanlon C, Medhin G, Fekadu A. Antenatal predictors of incident and persistent postnatal depressive symptoms in rural Ethiopia: A population-based prospective study. Reprod Health. 2019;16(1):1–9.

29. World Health Organization (WHO). Caring for women subjected to violence: a WHO curriculum for training health-care providers. Geneva; 2019.

30. Decker MR, Wood SN, Hameeduddin Z, Kennedy SR, Perrin N, Tallam C, et al. Safety decision-making and planning mobile app for intimate partner violence prevention and response: randomised controlled trial in Kenya. BMJ Glob Heal. 2020;5(002091).

For peer review only - http://bmjopen.bmj.com/site/about/guidelines.xhtml

Table 1. Characteristics of 6-Week Interview Participants by Exposure (n=2,388)

Demographic Characteristics	Pre-COVID 6-Week Interview (n=1,405)	During-COVID 6-Week Interview (n=983)	p-value
	n (row %)		
Region			0.28
Tigray	107 (61.5)	66 (38.5)	
Afar	23 (50.0)	23 (50.0)	
Amhara	305 (61.4)	191 (38.6)	
Oromiya	573 (55.6)	459 (44.4)	
SNNP	332 (59.6)	225 (40.4)	
Addis Ababa	45 (54.4)	38 (45.6)	
Residence			0.14
Urban	320 (61.0)	205 (39.0)	
Rural	1064 (57.2)	797 (42.8)	
Household wealth			0.23
Lower	531 (56.2)	413 (43.8)	
Higher	854 (58.2)	589 (40.8)	
Age			0.86
15-19	147 (56.5)	114 (43.6)	
20-29	736 (57.9)	534 (42.1)	
30-49	502 (58.6)	355 (41.4)	
Parity			0.001
Nulliparous	243 (50.3)	238 (49.5)	
1-2	538 (62.3)	326 (37.7)	
3+	604 (57.9)	438 (42.1)	
Education			0.95
Never attended	579 (57.8)	422 (42.2)	
Primary	554 (58.5)	393 (41.5)	
Secondary or higher	252 (57.4)	187 (42.6)	

For peer review only

Table 2. Bivariate and Logistic Regression Examining Type of Violence Post-COVID, Compared to Pre-COVID, Overall and by Residence

Type of Violence	Overall (n=2,388)			Urban (n=884)			Rural (n=1,504)		
	Pre (n=1,405)	During (n=983)	aOR† (95% CI)	Pre (n=540)	During (n=344)	aOR¥ (95% CI)	Pre (n=865)	During (n=639)	aOR¥ (95% CI)
	n (%)			n (%)			n (%)		
Any IPV	147 (10.5)	149 (15.1)	1.51* (1.06, 2.15)	30 (5.6)	37 (10.9)	2.09* (1.10, 3.96)	103 (11.9)	103 (16.2)	1.43 ± (0.96, 2.13)
Physical Violence	70 (5.0)	76 (7.8)	1.60* (1.07, 2.40)	19 (3.5)	27 (7.9)	2.46 * (1.18, 5.10)	47 (5.4)	49 (7.7)	1.46 (0.90, 2.36)
Sexual Violence	104 (7.4)	96 (9.8)	1.33 (0.88, 2.02)	15 (2.8)	16 (4.7)	1.70 (0.59, 4.91)	76 (8.8)	71 (11.1)	1.30 (0.83, 2.03)

†aOR adjusted for residence, age, and education; parity omitted for multicollinearity
¥aOR adjusted for age and education; parity omitted for multicollinearity
±p<0.10; *p<0.05; **p<0.01; p<0.001

For peer review only

Figure 1. Venn Diagram of Types of Violence Experienced During Pregnancy, by Pre/Post Exposure

For peer review only

For peer review only - http://bmjopen.bmj.com/site/about/guidelines.xhtml

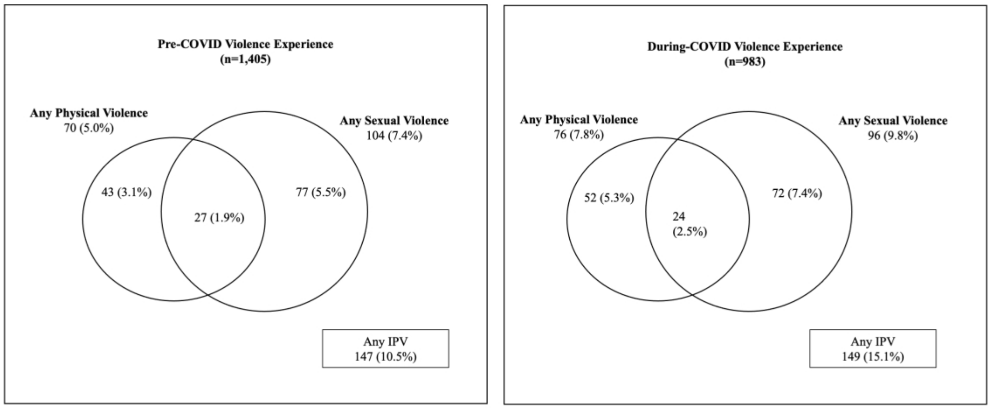

Figure 1. Venn Diagram of Types of Violence Experienced During Pregnancy, by Pre/Post Exposure

338x190mm (216 x 216 DPI)

STROBE Statement—Checklist of items that should be included in reports of ***cohort studies***

	Item No	Recommendation	Page No
**Title and abstract**	1	(*a*) Indicate the study's design with a commonly used term in the title or the abstract<br>(*b*) Provide in the abstract an informative and balanced summary of what was done and what was found	1
**1Introduction**			
Background/rationale	2	Explain the scientific background and rationale for the investigation being reported	4
Objectives	3	State specific objectives, including any prespecified hypotheses	4
**Methods**			
Study design	4	Present key elements of study design early in the paper	4
Setting	5	Describe the setting, locations, and relevant dates, including periods of recruitment, exposure, follow-up, and data collection	4
Participants	6	(*a*) Give the eligibility criteria, and the sources and methods of selection of participants. Describe methods of follow-up<br>(*b*) For matched studies, give matching criteria and number of exposed and unexposed	4
Variables	7	Clearly define all outcomes, exposures, predictors, potential confounders, and effect modifiers. Give diagnostic criteria, if applicable	5
Data sources/ measurement	8*	For each variable of interest, give sources of data and details of methods of assessment (measurement). Describe comparability of assessment methods if there is more than one group	5
Bias	9	Describe any efforts to address potential sources of bias	6
Study size	10	Explain how the study size was arrived at	4
Quantitative variables	11	Explain how quantitative variables were handled in the analyses. If applicable, describe which groupings were chosen and why	5
Statistical methods	12	(*a*) Describe all statistical methods, including those used to control for confounding<br>(*b*) Describe any methods used to examine subgroups and interactions<br>(*c*) Explain how missing data were addressed<br>(*d*) If applicable, explain how loss to follow-up was addressed<br>(*e*) Describe any sensitivity analyses	5
**Results**			
Participants	13*	(a) Report numbers of individuals at each stage of study—eg numbers potentially eligible, examined for eligibility, confirmed eligible, included in the study, completing follow-up, and analysed<br>(b) Give reasons for non-participation at each stage<br>(c) Consider use of a flow diagram	4
Descriptive data	14*	(a) Give characteristics of study participants (eg demographic, clinical, social) and information on exposures and potential confounders<br>(b) Indicate number of participants with missing data for each variable of interest<br>(c) Summarise follow-up time (eg, average and total amount)	6
Outcome data	15*	Report numbers of outcome events or summary measures over time	7

For peer review only - http://bmjopen.bmj.com/site/about/guidelines.xhtml

Main results	16	(*a*) Give unadjusted estimates and, if applicable, confounder-adjusted estimates and their precision (eg, 95% confidence interval). Make clear which confounders were adjusted for and why they were included<br>(*b*) Report category boundaries when continuous variables were categorized<br>(*c*) If relevant, consider translating estimates of relative risk into absolute risk for a meaningful time period	7
Other analyses	17	Report other analyses done—eg analyses of subgroups and interactions, and sensitivity analyses	7,8
**Discussion**			
Key results	18	Summarise key results with reference to study objectives	8
Limitations	19	Discuss limitations of the study, taking into account sources of potential bias or imprecision. Discuss both direction and magnitude of any potential bias	9
Interpretation	20	Give a cautious overall interpretation of results considering objectives, limitations, multiplicity of analyses, results from similar studies, and other relevant evidence	8,9
Generalisability	21	Discuss the generalisability (external validity) of the study results	9
**Other information**			
Funding	22	Give the source of funding and the role of the funders for the present study and, if applicable, for the original study on which the present article is based	1

*Give information separately for exposed and unexposed groups.

Note: An Explanation and Elaboration article discusses each checklist item and gives methodological background and published examples of transparent reporting. The STROBE checklist is best used in conjunction with this article (freely available on the Web sites of PLoS Medicine at http://www.plosmedicine.org/, Annals of Internal Medicine at http://www.annals.org/, and Epidemiology at http://www.epidem.com/). Information on the STROBE Initiative is available at http://www.strobe-statement.org.

For peer review only

BMJ Open

Impact of the COVID-19 pandemic on intimate partner violence during pregnancy: evidence from a multi-methods study of recently pregnant women in Ethiopia

Journal:	*BMJ Open*
Manuscript ID	bmjopen-2021-055790.R1
Article Type:	Original research
Date Submitted by the Author:	27-Jan-2022
Complete List of Authors:	Wood, Shannon; Johns Hopkins University Bloomberg School of Public Health<br>Yirgu, Robel; Addis Ababa University<br>Wondimagegnehu, Abigiya; Addis Ababa University<br>Qian, Jiage; Johns Hopkins University Bloomberg School of Public Health<br>Milkovich, Rachel; Johns Hopkins University Bloomberg School of Public Health<br>Decker, Michele; Johns Hopkins University Bloomberg School of Public Health<br>Glass, Nancy; Johns Hopkins University School of Nursing<br>Seid, Fatuma; Ethiopia Ministry of Health<br>Zekarias, Lensa; Ethiopia Ministry of Health<br>Zimmerman, Linnea; Johns Hopkins University Bloomberg School of Public Health, Population Family and Reproductive Health
**Primary Subject Heading**:	Global health
Secondary Subject Heading:	Respiratory medicine
Keywords:	COVID-19, Public health < INFECTIOUS DISEASES, QUALITATIVE RESEARCH, PUBLIC HEALTH, REPRODUCTIVE MEDICINE, SEXUAL MEDICINE

SCHOLARONE™
Manuscripts

I, the Submitting Author has the right to grant and does grant on behalf of all authors of the Work (as defined in the below author licence), an exclusive licence and/or a non-exclusive licence for contributions from authors who are: i) UK Crown employees; ii) where BMJ has agreed a CC-BY licence shall apply, and/or iii) in accordance with the terms applicable for US Federal Government officers or employees acting as part of their official duties; on a worldwide, perpetual, irrevocable, royalty-free basis to BMJ Publishing Group Ltd ("BMJ") its licensees and where the relevant Journal is co-owned by BMJ to the co-owners of the Journal, to publish the Work in this journal and any other BMJ products and to exploit all rights, as set out in our licence.

The Submitting Author accepts and understands that any supply made under these terms is made by BMJ to the Submitting Author unless you are acting as an employee on behalf of your employer or a postgraduate student of an affiliated institution which is paying any applicable article publishing charge ("APC") for Open Access articles. Where the Submitting Author wishes to make the Work available on an Open Access basis (and intends to pay the relevant APC), the terms of reuse of such Open Access shall be governed by a Creative Commons licence – details of these licences and which Creative Commons licence will apply to this Work are set out in our licence referred to above.

Other than as permitted in any relevant BMJ Author's Self Archiving Policies, I confirm this Work has not been accepted for publication elsewhere, is not being considered for publication elsewhere and does not duplicate material already published. I confirm all authors consent to publication of this Work and authorise the granting of this licence.

For peer review only

Title: Impact of the COVID-19 pandemic on intimate partner violence during pregnancy: evidence from a multi-methods study of recently pregnant women in Ethiopia

Authorship team: Shannon N. Wood[1], Robel Yirgu[2], Abigiya Wondimagegnehu[2], Jiage Qian[1], Rachel Milkovich[1], Michele R. Decker[1,3,4], Nancy Glass[4,5], Fatuma Seid[6], Lensa Zekarias[6], Linnea A. Zimmerman[1]

Affiliations:
[1] Department of Population Family and Reproductive Health, Johns Hopkins Bloomberg School of Public Health, Baltimore, Maryland, USA.
[2] Addis Ababa University School of Public Health, Addis Ababa, Ethiopia.
[3] Center for Public Health and Human Rights, Johns Hopkins Bloomberg School of Public Health, Baltimore, Maryland, USA.
[4] Johns Hopkins School of Nursing, Baltimore, Maryland, USA.
[5] Center for Global Health, Johns Hopkins University, Baltimore, Maryland, USA.
[6] Ethiopia Federal Ministry of Health, Addis Ababa, Ethiopia.

Corresponding Author:
Shannon N. Wood
615 N. Wolfe Street
Baltimore, MD
swood@jhu.edu

Word count: 3,151
Figures: 1
Tables: 2

For peer review only - http://bmjopen.bmj.com/site/about/guidelines.xhtml

Abstract

Objectives: This multi-methods study aimed to: 1) compare the prevalence of intimate partner violence (IPV) during pregnancy pre- and during the COVID-19 pandemic using quantitative data; and 2) contextualize pregnant women's IPV experiences during the COVID-19 pandemic through supplemental interviews.

Design: Quantitative analyses utilize data from Performance Monitoring for Action (PMA)-Ethiopia, a cohort of 2,873 pregnant women that collects data at pregnancy, 6-weeks, 6-months, and 1-year postpartum. Following 6-week postpartum survey, in-depth semi-structured interviews contextualized experiences of IPV during pregnancy with a subset of participants (n=24).

Participants: All pregnant women residing within six regions of Ethiopia, covering 91% of the population, were eligible for the cohort study (n=2,868 completed baseline survey). Quantitative analyses were restricted to the 2,388 women with complete six-week survey data (retention=82.7%). A purposive sampling frame was used to select qualitative participants on baseline survey data, with inclusion criteria specifying completion of quantitative 6-week interview after the onset of the COVID-19 pandemic, and indication of IPV experience.

Interventions: A State of Emergency in Ethiopia was declared in response to the COVID-19 pandemic approximately halfway through 6-week postpartum interview, enabling a natural experiment (n=1,405 pre-COVID-19; n=983 during-COVID-19).

Primary outcome measures: IPV during pregnancy was assessed via the 10-item Revised Conflict and Tactics Scale (CTS-2).

Results: One in ten women experienced any IPV during pregnancy prior to COVID-19 (10.5%), and prevalence of IPV during pregnancy increased to 15.1% during the COVID-19 pandemic (aOR=1.51; p=0.02). Stratified by residence, odds of IPV during the pandemic increased for urban women only (aOR=2.09; p=0.03), however, IPV prevalence was higher in rural regions at both timepoints. Qualitative data reveal COVID-19-related stressors namely, loss of household income and increased time spent within the household, exacerbated IPV.

Conclusions: These multi-methods results highlight the prevalent, severe violence that pregnant Ethiopian women experience, with pandemic-related increases concentrated in urban areas. Integration of IPV response and safety planning across the continuum of care can mitigate impact.

For peer review only

Article summary

Strengths and limitations of this study

- Natural experiment based on split of pre-existing cohort to examine differences in intimate partner violence (IPV) during pregnancy pre/post COVID-19 restrictions
- Large quantitative sample inclusive of all pregnant women residing within six regions of Ethiopia, covering 91% of the population
- In-depth interviews to contextualize women's experiences with IPV during pregnancy during the COVID-19 pandemic
- Groupings may not exactly estimate pre/post exposure and no women who were truly only exposed to IPV during the COVID-19 pandemic
- Qualitative interviews only occurred within two regions and are not transferable to all pregnant women experiencing IPV during pregnancy

For peer review only

Introduction

Pandemics exacerbate gender inequities, including intimate partner violence (IPV) (1–3). Evidence from previous epidemics and regional crises indicates heightened IPV via increased economic insecurity, social isolation, and exposure to perpetrators, and more limited options for garnering support (1). COVID-19 is no exception—12 out of 15 early studies indicate increases in IPV globally (4).

The pregnancy and postpartum period are pivotal timepoints for assessing and responding to IPV, given health impacts to mother and baby, and multiple points of contact with healthcare providers. Global estimates indicate that 2-14% of women experience violence during pregnancy (5,6). Pre-existing IPV may be sustained or exacerbated, and abusive partners may limit access to antenatal and postnatal care (6). IPV during pregnancy incurs profound effects, including miscarriage, premature labor, low birthweight, and maternal depression (7–10). Timely response is thus critical for this vulnerable sub-population (5,10). Linkages to woman-centered care may decrease subsequent abuse (10).

Violence prevention and response is critical within the Ethiopian context. Ethiopia Demographic and Health Survey (DHS) data indicate that 27% of ever-married women age 15-49 have experienced IPV within the past year and 4% experienced IPV during pregnancy (11). In April 2020, Ethiopia declared a State of Emergency in response to the COVID-19 pandemic, with resultant lockdown measures including, but not limited to, physical distancing, school closures, prohibition of in-house visitation outside of family members, capacity limits and masking requirements on public transportation, and discouragement of within-country travel (12); these measures may disproportionately impact urban residents due to population density, urban planning, and health-seeking behaviors (13). Further, though essential to limit infection, such lockdown measures may exacerbate pregnant women's IPV experiences (2,3). Early evidence from Amhara, Ethiopia indicated similar levels of IPV pre- and during COVID-19 (14), while 18% of antenatal care attendees in Addis Ababa reported perceived increase in violence during pregnancy since the onset of the pandemic (15). Both studies, however, were limited in generalizability due to cross-sectional design and sampling considerations.

While global evidence indicates increases in IPV, to date, no studies aimed at understanding the impact of the COVID-19 pandemic or related response measures have examined violence during pregnancy within the Ethiopian context (4). Ethical standards advocate for continued IPV monitoring using existing study infrastructures if the research aims to understand the magnitude of IPV burden and linkages with social/economic factors; informs response efforts; and meets ethical obligations (1,16). Utilizing an existing national cohort of recently pregnant women and rigorous ethical standards, this study aimed to examine how the COVID-19 pandemic affected pregnant women's experiences with IPV through 1) comparison of IPV prevalence pre- and post-COVID-19 using quantitative data and 2) contextualization of women's experiences of IPV during the COVID-19 pandemic.

Methods

Overall Study Design

This multi-methods analysis is situated within the Performance Monitoring for Action (PMA)-Ethiopia cohort study, a collaboration between Johns Hopkins Bloomberg School of Public Health (JHSPH), Addis Ababa University (AAU), and the Ethiopian Federal Ministry of Health (FMoH). PMA-Ethiopia collects quantitative data on a cohort of 2,879 pregnant women at pregnancy (any gestation), 6-weeks, 6-months, and 1-year postpartum. Enrollment into the cohort began in October 2019. The full protocol for PMA Ethiopia is detailed elsewhere (17). The present analysis utilizes quantitative 6-week postpartum data to allow for most comprehensive measurement of violence throughout the entire pregnancy period. Following 6-week interview, in-depth interviews contextualized experiences of IPV during pregnancy, among a subset of participants (n=24); the qualitative phase was not a component of the original quantitative study design, and the quantitative data did not inform qualitative analyses, and vice versa. Institutional Review Board

For peer review only - http://bmjopen.bmj.com/site/about/guidelines.xhtml

approval was obtained at both JHSPH (IRB00013278) and AAU College of Health Sciences (077/20/SPH), and protocols were implemented in line with best practices for violence research (18).

Quantitative Participants

All pregnant women residing within six regions of Ethiopia that covers 91% of the population were eligible for the cohort study (n=2,868 completed baseline survey). Quantitative analyses were restricted to the 2,388 women with complete six-week survey data (retention=82.7%). A State of Emergency in Ethiopia was declared in response to the COVID-19 pandemic approximately halfway through fielding the 6-week postpartum interview (April 8, 2020), thus enabling a natural experiment. Specifically, 1,405 6-week interviews were conducted before the onset of the COVID-19 pandemic; the remaining 983 occurred after COVID-19 emergency lockdown procedures eased in early June and data collection was able to resume.

Quantitative Measures

Intimate partner violence, the primary outcome of interest, was measured via the 10-item Revised Conflict and Tactics Scale (19), which asks about specific violence behaviors at any time during pregnancy, per best practices for violence research (18).Three violence measures were examined: 1) any IPV, 2) any physical IPV, 3) any sexual IPV; affirmative response to any behavior was classified as IPV experience. Physical and sexual IPV were derived from the following items: 1) physical IPV: "push you, shake you, or throw something at you;" "slap you;" "twist your arm or pull your hair;" "punch you with his fist or with something that could hurt you;" "kick you, drag you, or beat you up;" "try to choke you or burn you on purpose;" "threaten or attack you with a knife, gun, or other weapon" and 2) sexual IPV: "physically force you to have sexual intercourse with him when you did not want to;" "physically force you to perform any other sexual acts you did not want to;" "used threats or pressure to make you have sex when you did not want to." Any physical and any sexual IPV were not mutually exclusive.

The primary exposure variable of interest measures pre/post exposure to COVID-19 restrictions, a binary variable, indicating whether a woman completed her 6-week postpartum survey before (pre-lockdown) or after (post-lockdown) April 8, when the State of Emergency was declared and data collection paused.

Analyses were stratified by urban/rural residence given the differences in COVID-19 lockdown measures. Sociodemographic variables explored as adjustment variables were chosen on a conceptual basis, and included region, household wealth, age, parity, and education; all were examined in categorical form.

Quantitative Analysis

Descriptive statistics examined the distribution of sociodemographic characteristics by pre/post exposure; design-based F statistics assessed whether distributions were similar. Venn diagrams classified violence experiences during each timepoint. Next, bivariate distributions of each type of violence were examined by exposure, overall and stratified by urban/rural residence. Bivariate (not presented) and multivariable logistic regression models were used to examine differences in violence experience pre/during the COVID-19 pandemic and residence; for adjusted models, correlations between covariates were examined for multi-collinearity, with only residence, age, and education retained within final models. All analyses were conducted in STATA 16, with statistical significance set at $p<0.05$, and accounting for complex-survey design.

Qualitative Participants

To contextualize the impact of the COVID-19 pandemic on women's experiences of violence during pregnancy, an explanatory qualitative phase was conducted following principles for qualitative descriptive design (20). Specifically, 24 purposively sampled semi-structured qualitative interviews focused on IPV were conducted in the month following 6-week quantitative data collection in Oromiya and SNNP regions; regions were selected based on high IPV during pregnancy from baseline survey and feasibility. At the 6-

For peer review only - http://bmjopen.bmj.com/site/about/guidelines.xhtml

week survey, participants were consented for potential follow-up specific to partner-related items. A purposive sampling frame was used to select participants on baseline survey data, with inclusion criteria specifying: 1) completion of quantitative 6-week post-COVID-19 interview; 2) indication of IPV experience via quantitative data. These eligibility criteria generated a sampling frame of 17 IPV survivors in Oromiya and 18 IPV survivors in SNNP. From this purposive sampling frame, participants were randomly selected and data collection continued until feasible sample size was met (n=14 Oromiya; n=10 SNNP) (21).

Qualitative Data Collection

Training for the qualitative phase preceded data collection with focus on probing, ethical principles for IPV research, and research team protections. Semi-structured interview guides focused on women's experiences with IPV and IPV services. Participants were called prior to interview for scheduling considerations. All interviews lasted approximately 25-30 minutes. Four trained interviewers used a structured note-taking tool to allow probing of experiences, while permitting rapid analysis for timely results (22). Interviews were conducted in Amharic, Afan Oromo, or local languages of SNNP with the help of local translators. Immediately post-interview, interviewers typed and translated field notes.

Qualitative Analysis

Two researchers trained in qualitative analysis coded 24 structured notes using Atlas.ti software. Inductive thematic analysis was used to identify emergent themes and sub-themes and to create an initial set of codes. Dual coding and retro-coding were used to enhance agreement between coders. Coders also met and collaborated regularly with the field research team to discuss and clarify interpretation. Coding was complete when saturation of themes was achieved (23); illustrative quotes were then downloaded from Atlas.ti and organized in matrices of code themes that were organized by IPV experience.

Patient and Public Involvement

Community members are not directly involved in PMA-Ethiopia research, however, a Project Advisory Board, including members of the Federal Ministry of Health, health providers, and key stakeholders, are consulted about country-specific priorities, including IPV, during survey development. Results are disseminated to the advisory board to inform action.

Results

Quantitative Results

Demographic characteristics of study participants by pre/post exposure are presented in Table 1. Significant differences were observed for parity only, where women of higher parity were more likely to be interviewed pre-COVID-19.

Figure 1 displays overlap of types of violence experienced by women during pregnancy, by exposure. At both timepoints, sexual IPV (7.4%$_{\text{pre-COVID-19}}$; 9.8%$_{\text{during-COVID-19}}$) was higher than physical IPV (5.0%$_{\text{pre-COVID-19}}$; 7.8%$_{\text{during-COVID-19}}$) and often occurred in isolation.

[insert Figure 1 here]

Bivariate and logistic regression results comparing pre-COVID-19 IPV to during COVID-19 IPV, overall and by residence, are presented in Table 2. One in ten women experienced any IPV during pregnancy prior to COVID019 (10.5%), and this proportion increased to 15.1% during the COVID-19 pandemic (aOR during COVID-19 compared to pre-COVID-19=1.51; 95% CI=1.06-2.15; p=0.02). When stratified by residence, odds of IPV during COVID-19, compared to pre-COVID-19, increased for urban women (aOR=2.09; 95%CI=1.10-3.96; p=0.03), while odds slightly attenuated for rural women, but the difference did not achieve statistical significance (aOR=1.43; 95% CI=0.96-2.13; p=0.08). IPV prevalence was

For peer review only - http://bmjopen.bmj.com/site/about/guidelines.xhtml

consistently higher among rural women at both time points (16.2% during COVID-19, 11.9% pre-COVID-19 for rural women vs. 10.9% during COVID-19, 5.6% pre-COVID-19 for urban women).

In examining violence sub-types, physical IPV increased 60% during COVID-19, compared to pre-COVID-19, in the overall sample (aOR=1.60; 95% CI=1.07-2.40; p=0.02); this increase was greater than two-fold in urban areas (aOR=2.46; 95% CI=1.18-5.10; p=0.02). Statistically significant changes by pre/post exposure were not observed for physical violence in rural areas or for sexual violence.

Qualitative Results

Qualitative data indicate that the COVID-19 pandemic and implementation of local lockdown measures affected IPV within relationships differentially based on husband's job status. Specifically, women whose husbands experienced loss of income or employment due to the pandemic often reported that the COVID-19 pandemic put stress on their relationship and increased instances of physical violence. These stressors were described by an 18-year-old woman in Oromiya who had just given birth to her first child:

> Since he was not working, he didn't have money; it was difficult for us to pay the house rent, it was difficult to buy food. These problems created conflict, then he insulted and hit me.

Further, many women experienced an increase in verbal abuse due to COVID-19-related stressors. Several participants shared that the main reason for conflict prior to the pandemic were disagreements about household responsibilities, suggesting that the COVID-19 pandemic exacerbated this tension. This increase in household tension due to restrictions on movement was discussed by a 36-year-old woman with four children in rural SNNP:

> The violence increased because we stayed together at our house because there was movement restriction in our village so he couldn't go to the market to sell goats and sheep. So, our income decreased a lot and because of that we had arguments most of the time.

The pandemic also changed the amount of time that spouses spent at home together, most often due to a husband's loss of employment. One 29-year-old participant from rural Oromiya disclosed that this gave her husband an opportunity to further control her activities and to engage in sexual violence at a high frequency:

> During corona time, he stays at home so he is happy with that because he can control all my day-to-day activities. No physical and psychological violence happened but sometimes there is sexual violence. It might be four times a week.

It should be noted that several participants stated that the pandemic had little or no impact on their relationships with their husbands, however, previous violence persisted throughout. An example of this endemic violence is described by a 37-year-old IPV survivor from rural Oromiya:

> We had a disagreement during this pregnancy. It was during coronavirus. But the cause was not related with corona. We disagreed due to children. The corona pandemic has nothing to do with our relationship.

In many instances, the violence described by women during pregnancy was severe—several examples of violence severity, particularly in relation to physical violence, are described below.

> When I became pregnant, the part of my body he hits was changed but not the frequency. He used to beat every part of my body but when I became pregnant, he especially didn't hit me around the belly. Because he cares for the baby. He slaps my face with my own hand. -36-year-old IPV survivor with four children from rural SNNP

For peer review only - http://bmjopen.bmj.com/site/about/guidelines.xhtml

He doesn't care if I am pregnant or not. When he is very angry, he gets very emotional and throws at me whatever he finds, whether it is a chair or anything. I had stillbirth, the fetus died in the belly, though I had a spontaneous vaginal delivery. When he heard the baby died in my uterus, he got angry and thought I was the one who killed the baby. -18-year-old nulliparous IPV survivor from rural SNNP

When I was nine months pregnant; he hits me and throw me on the stone then I went to my father's home. Immediately, my father's neighbors took me to hospital. I was admitted there for 15 days. -18-year-old primiparous IPV survivor from urban Oromiya

Discussion

These multi-methods results highlight the prevalent, severe violence that women experience at a critical, and often vulnerable, period during their reproductive lives. Utilizing a natural experiment embedded in cohort of recently pregnant women in the six largest regions of Ethiopia, we found heightened IPV during the COVID-19 pandemic, compared to the pre-COVID-19 period, with greatest between-time impact concentrated in urban areas. Discrepancies by residence corroborate heightened cases and more COVID-19 preventive measures within urban areas (12). Qualitative data reveal the mechanisms through which pandemic-related stressors operate to exacerbate IPV, namely loss of household income and increased time spent within the household.

More than one in ten women experienced violence during pregnancy within Ethiopia ($10.5\%_{\text{pre-COVID-19}}$)—IPV during pregnancy reported by recently pregnant women in this study was substantially higher than 2016 DHS lifetime estimates (3.7%) (11). Consistent with the World Health Organization Multi-Country Study on Women's Health and Domestic Violence and DHS (6,11), women reported higher levels of sexual violence than physical violence—notably, Ethiopia is one of the few contexts globally where this pattern is observed. Continuous monitoring within national surveillance systems and large-scale cohort studies can assist identifying who is at risk for IPV during most recent pregnancy. Utilizing best practices for violence-related research (16,18), e.g. timely linkage to woman-centered referrals, can bolster safety and minimize adverse outcomes.

Given distinct patterns of IPV by urban and rural residence, interventions must be tailored by locality and women's circumstances. The impact of the COVID-19 pandemic on violence during pregnancy was concentrated primarily within urban settings. Immediate intervention and connection to IPV referral services should focus on urban settings, prone to closures and economic impact. As time and household finances were indicated as stressors, temporary separation and seeking support from family members and neighbors may be a feasible strategy to minimize impact of violence—this safety strategy has been useful for women experiencing IPV in urban informal settlements of Nairobi and other low- and middle-income contexts where leaving the relationship is not feasible or socially acceptable (24,25). Notably, however, IPV during pregnancy was highest for rural women at both timepoints ($16.2\%_{\text{rural}}$ vs. $10.9\%_{\text{urban}}$ during COVID-19). Heightened prevalence within rural settings likely speaks to cohesive, patriarchal community norms promoting violence against women (26); in order to ultimately reduce IPV, large-scale transformative community norms interventions, such as Communities Care or SASA! (27,28), will be required.

This study is not without limitations—namely, these groupings may not exactly estimate pre/post exposure and we have no women who were truly only exposed to IPV during the COVID-19 pandemic. Further, our sample size was limited for additional subgroup analysis to examine women most at risk for IPV during pregnancy by pre-COVID-19 and during-COVID-19 time points. To maximize women's confidentiality and bolster safety in line with best practices for violence-related research (18), interviewers were instructed to not conduct either quantitative or qualitative interviews within the presence of a partner; given COVID-

For peer review only - http://bmjopen.bmj.com/site/about/guidelines.xhtml

19 lockdown measures and potential for controlling behaviors within abusive partnerships, our results may be an underestimate. Lastly, qualitative data collection occurred only within two of the six regions based on baseline prevalence of IPV during pregnancy—this purposive sampling strategy may have excluded women needing IPV support during the COVID-19 pandemic and is not transferable to all pregnant women experiencing IPV in Ethiopia.

While IPV during pregnancy increased overall during the COVID-19 pandemic, violence and related gender-based power disparities were prominent prior to the onset of the pandemic—accordingly, IPV prevention and response efforts in Ethiopia must be sustained in the post-pandemic era. We offer two concrete recommendations for maternal health providers. First, while antenatal care is a critical intervention point for identifying and providing care for women experiencing IPV during pregnancy, providers across the maternal and neonatal continuum of care must similarly provide support. Integration of IPV screening and psychological care into postnatal care is critical given links to postpartum depression (29,30). Clinic-based aids, including World Health Organization recommendations for clinical IPV identification and linkage to health and economic referrals, can be valuable in training providers to support survivors (31). Second, the use of safety decision-aids by providers across the continuum of care could assist in helping women assess their circumstances and level of danger, and create safety plans tailored to their situations. Safety planning with trained community health workers was found to increase safety preparedness in Kenya (32), and may be similarly valuable in rural areas of Ethiopia with trained Health Extension Workers. Clinic-based interventions to reduce and address IPV should not occur separately from other health services, but as part of larger community-led behavioral change programs with influential community members on targeting harmful gender norms and IPV, in order to ultimately empower women and girls. The COVID-19 pandemic has alerted the global community to pervasiveness of violence against women—continued momentum and investment is needed to mitigate its harmful impact.

For peer review only

Contributorship Statement: SNW designed the study, oversaw study details, and drafted the manuscript. RY oversaw all aspects of qualitative training and data collection. AW conducted training and data collection. JQ and RM conducted qualitative coding. MRD and NG served as ethical supervisors in the project. FS and LZ provided technical insight into Ethiopian IPV response systems. LAZ served as the Principal Investigator for the parent study and provided technical assistance. All authors participated in writing and approving the final manuscript.

Competing Interests: None declared.

Funding: Bill and Melinda Gates Foundation [INV 009466]and the Johns Hopkins Specialized Center for Research Excellence in Sex Differences (U54AG062333). Under the grant conditions of the Bill and Melinda Gates Foundation, a Creative Commons Attribution 4.0 Generic License has already been assigned to the Author Accepted Manuscript version that might arise from this submission.

Data Sharing Agreement: Quantitative data are available upon request from pmadata.org. Qualitative data are not available to maximize participant confidentiality.

Ethics Approval Statement: Johns Hopkins Bloomberg School of Public Health (IRB00013278) and Addis Ababa University College of Health Sciences (077/20/SPH)

For peer review only

References

1. Peterman A, Potts A, Donnell MO, Shah N, Oertelt-Prigione S, Gelder N Van. Working Paper 528: Pandemics and Violence Against Women and Children. Washington, DC;
2. Gelder N Van, Peterman A, Potts A, Donnell MO, Thompson K, Shah N, et al. COVID-19: Reducing the risk of infection might increase the risk of intimate partner violence. EClinicalMedicine. 2020;21.
3. Roesch E, Amin A, Gupta J, Garcia-Moreno C. Violence against women during covid-19 pandemic restrictions. BMJ. 2020;369(m1712):1–2.
4. Bourgault S, Peterman A, O'Donnell M. Violence Against Women and Children During COVID-19 — One Year On and 100 Papers In: A Fourth Research Round Up. 2021.
5. Devries KM, Kishor S, Johnson H, Stöckl H, Bacchus LJ, Garcia-Moreno C, et al. Intimate partner violence during pregnancy: analysis of prevalence data from 19 countries. Reprod Health Matters. 2010;18(36):158–70.
6. García-Moreno C, Jansen HA, Ellsberg M, Heise L, Watts C. WHO multi-country study on women's health and domestic violence against women. Geneva; 2005.
7. Hill A, Pallitto C, Mccleary-Sills J, Garcia-moreno C. A systematic review and meta-analysis of intimate partner violence during pregnancy and selected birth outcomes. Int J Gynecol Obstet. 2016;133:269–76.
8. Alhusen JL, Ray E, Sharps P, Bullock L. Intimate Partner Violence During Pregnancy: Maternal and Neonatal Outcomes. J Women. 2015;24(1):100–6.
9. Berhanie E, Gebregziabher D, Berihu H, Gerezgiher A, Kidane G. Intimate partner violence during pregnancy and adverse birth outcomes : a case-control study. 2019;1–9.
10. Chisholm C, Bullock L, Ferguson II JE. Intimate partner violence and pregnancy: epidemiology and impact. Am J Obstet Gynecol. 2017;(August):141–4.
11. Central Statistical Agency and ICF. Ethiopia Demographic and Health Survey. Addis Ababa, Ethiopia and Rockville, Maryland, USA; 2016.
12. Our World in Data. Policy Responses to the Coronavirus Pandemic--Statistics and Research [Internet]. [cited 2022 Jan 26]. Available from: https://ourworldindata.org/policy-responses-covid
13. Zimmerman LA, Desta S, Karp C, Yihdego M, Seme A, Shiferaw S, et al. Effect of the COVID-19 pandemic on health facility delivery in Ethiopia ; results from PMA Ethiopia's longitudinal panel. PLOS Glob Public Heal. 2021;1–14.
14. Tadesse AW, Tarekegn SM, Wagaw GB, Muluneh MD, Kassa AM. Prevalence and Associated Factors of Intimate Partner Violence Among Married Women During COVID-19 Pandemic Restrictions: A Community-Based Study. J Interpers Violence. 2020;
15. Teshome A, Gudu W, Bekele D, Asfaw M, Enyew R, Compton SD. Intimate partner violence among prenatal care attendees amidst the COVID-19 crisis: The incidence in Ethiopia. Int J Gynecol Obstet. 2021;153(1):45–50.
16. Peterman A, Bhatia A, Guedes A. Remote data collection on violence against women during COVID-19: A conversation with experts on ethics, measurement & research priorities.
17. Zimmerman L, Desta S, Yihdego M, Rogers A, Amogne A, Karp C, et al. Protocol for PMA-Ethiopia: A New Data Source for Cross-sectional and Longitudinal Data of Reproductive, Maternal, and Newborn Health. Accept Glob Heal Sci Pract. 2020;
18. World Health Organization (WHO). Ethical and safety recommendations for intervention research on violence against women. Geneva; 2016.
19. Straus M, Hamby S, Boney-McCoy S, Sugarman D. The Revised Conflict Tactics Scales (CTS2). J Fam Issues. 1996;17(3):283–316.
20. Bradshaw C, Atkinson S, Doody O. Employing a Qualitative Description Approach in Health Care Research. Glob Qual Nurs Res. 2017;4.
21. Guest G, Bunce A, Johnson L. How Many Interviews Are Enough? An Experiment with Data Saturation and Variability. Field methods. 2006;18(1):59–82.
22. Johnson BD, Dunlap E, Benoit E. Structured Qualitative Resesarch: Organizing "Mountains of

For peer review only

Words" for Data Analysis, both Qualitative and Quantitative. Subst Use Misuse. 2010;45(5):648–70.
23. Guest G, MacQueen K, Namey E. Applied thematic analysis. Thousand Oaks: Sage Publications; 2012.
24. Wood SN, Kennedy RS, Hameeduddin Z, Asira B, Tallam C, Akumu I, et al. "Being married doesn't mean you have to reach the end of the world:" Safety planning with intimate partner violence survivors and service providers in three urban informal settlements in Nairobi, Kenya. J Interpers Violence. 2019;Epub ahead of print.
25. Wood SN, Glass N, Decker MR. An integrative review of safety strategies for women experiencing intimate partner violence in low- and middle-income countries. Trauma, Violence, Abus. 2019;1–15.
26. Murphy M, Jones N, Yadete W, Baird S. Gender-norms, violence and adolescence: Exploring how gender norms are associated with experiences of childhood violence among young adolescents in Ethiopia. Glob Public Health. 2021;16(6):842–55.
27. Abramsky T, Devries K, Kiss L, Nakuti J, Kyegombe N, Starmann E, et al. Findings from the SASA ! Study : a cluster randomized controlled trial to assess the impact of a community mobilization intervention to prevent violence against women and reduce HIV risk in Kampala , Uganda. 2014;1–17.
28. Glass N, Perrin N, Clough A, Desgroppes A, Kaburu FN, Melton J, et al. Evaluating the communities care program: Best practice for rigorous research to evaluate gender based violence prevention and response programs in humanitarian settings. Confl Health. 2018;12(5):1–10.
29. Ashenafi W, Mengistie B, Egata G, Berhane Y. The role of intimate partner violence victimization during pregnancy on maternal postpartum depression in Eastern Ethiopia. SAGE Open Med. 2021;9:1–12.
30. Bitew T, Hanlon C, Medhin G, Fekadu A. Antenatal predictors of incident and persistent postnatal depressive symptoms in rural Ethiopia: A population-based prospective study. Reprod Health. 2019;16(1):1–9.
31. World Health Organization (WHO). Caring for women subjected to violence: a WHO curriculum for training health-care providers. Geneva; 2019.
32. Decker MR, Wood SN, Hameeduddin Z, Kennedy SR, Perrin N, Tallam C, et al. Safety decision-making and planning mobile app for intimate partner violence prevention and response: randomised controlled trial in Kenya. BMJ Glob Heal. 2020;5(002091).

For peer review only

1 **Table 1. Characteristics of 6-Week Interview Participants by Exposure (n=2,388)**

Demographic Characteristics	Pre-COVID-19 6-Week Interview (n=1,405)	During-COVID-19 6-Week Interview (n=983)	p-value
	n (row %)		
Region			0.28
Tigray	107 (61.5)	66 (38.5)	
Afar	23 (50.0)	23 (50.0)	
Amhara	305 (61.4)	191 (38.6)	
Oromiya	573 (55.6)	459 (44.4)	
SNNP	332 (59.6)	225 (40.4)	
Addis Ababa	45 (54.4)	38 (45.6)	
Residence			0.14
Urban	320 (61.0)	205 (39.0)	
Rural	1064 (57.2)	797 (42.8)	
Household wealth			0.23
Lower	531 (56.2)	413 (43.8)	
Higher	854 (58.2)	589 (40.8)	
Age			0.86
15-19	147 (56.5)	114 (43.6)	
20-29	736 (57.9)	534 (42.1)	
30-49	502 (58.6)	355 (41.4)	
Parity			0.001
Nulliparous	243 (50.3)	238 (49.5)	
1-2	538 (62.3)	326 (37.7)	
3+	604 (57.9)	438 (42.1)	
Education			0.95
Never attended	579 (57.8)	422 (42.2)	
Primary	554 (58.5)	393 (41.5)	
Secondary or higher	252 (57.4)	187 (42.6)	

2

3

For peer review only - http://bmjopen.bmj.com/site/about/guidelines.xhtml

Table 2. Bivariate and Logistic Regression Examining Type of Violence Post-COVID-19, Compared to Pre-COVID-19, Overall and by Residence

Type of Violence	Overall (n=2,388)			Urban (n=884)			Rural (n=1,504)		
	Pre (n=1,405)	During (n=983)	aOR† (95% CI)	Pre (n=540)	During (n=344)	aOR¥ (95% CI)	Pre (n=865)	During (n=639)	aOR¥ (95% CI)
	n (%)			n (%)			n (%)		
Any IPV	147 (10.5)	149 (15.1)	1.51* (1.06, 2.15)	30 (5.6)	37 (10.9)	2.09* (1.10, 3.96)	103 (11.9)	103 (16.2)	1.43 ± (0.96, 2.13)
Physical Violence	70 (5.0)	76 (7.8)	1.60* (1.07, 2.40)	19 (3.5)	27 (7.9)	2.46 * (1.18, 5.10)	47 (5.4)	49 (7.7)	1.46 (0.90, 2.36)
Sexual Violence	104 (7.4)	96 (9.8)	1.33 (0.88, 2.02)	15 (2.8)	16 (4.7)	1.70 (0.59, 4.91)	76 (8.8)	71 (11.1)	1.30 (0.83, 2.03)

†aOR adjusted for residence, age, and education; parity omitted for multicollinearity
¥aOR adjusted for age and education; parity omitted for multicollinearity
±p<0.10; *p<0.05; **p<0.01; p<0.001

For peer review only

Figure 1. Venn Diagram of Types of Violence Experienced During Pregnancy, by Pre/Post Exposure

For peer review only

For peer review only - http://bmjopen.bmj.com/site/about/guidelines.xhtml

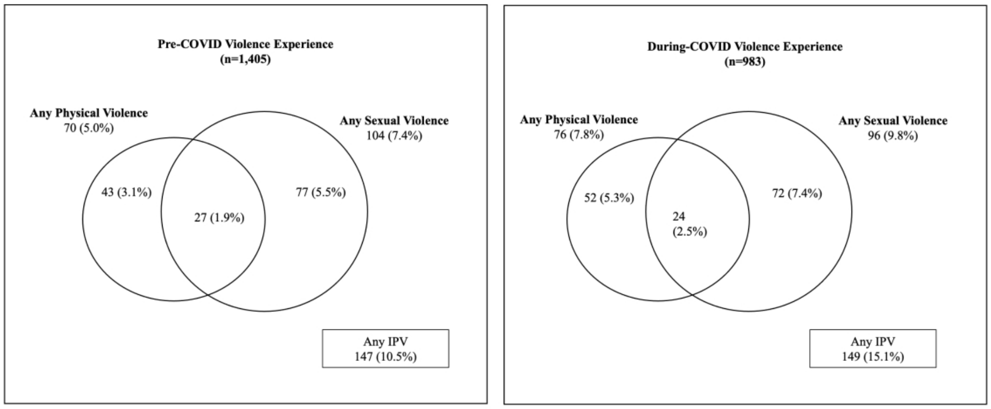

Figure 1. Venn Diagram of Types of Violence Experienced During Pregnancy, by Pre/Post Exposure

338x190mm (216 x 216 DPI)

STROBE Statement—Checklist of items that should be included in reports of ***cohort studies***

	Item No	Recommendation	Page No
**Title and abstract**	1	(*a*) Indicate the study's design with a commonly used term in the title or the abstract<br>(*b*) Provide in the abstract an informative and balanced summary of what was done and what was found	1
**1Introduction**			
Background/rationale	2	Explain the scientific background and rationale for the investigation being reported	4
Objectives	3	State specific objectives, including any prespecified hypotheses	4
**Methods**			
Study design	4	Present key elements of study design early in the paper	4
Setting	5	Describe the setting, locations, and relevant dates, including periods of recruitment, exposure, follow-up, and data collection	4
Participants	6	(*a*) Give the eligibility criteria, and the sources and methods of selection of participants. Describe methods of follow-up<br>(*b*) For matched studies, give matching criteria and number of exposed and unexposed	4
Variables	7	Clearly define all outcomes, exposures, predictors, potential confounders, and effect modifiers. Give diagnostic criteria, if applicable	5
Data sources/ measurement	8*	For each variable of interest, give sources of data and details of methods of assessment (measurement). Describe comparability of assessment methods if there is more than one group	5
Bias	9	Describe any efforts to address potential sources of bias	6
Study size	10	Explain how the study size was arrived at	4
Quantitative variables	11	Explain how quantitative variables were handled in the analyses. If applicable, describe which groupings were chosen and why	5
Statistical methods	12	(*a*) Describe all statistical methods, including those used to control for confounding<br>(*b*) Describe any methods used to examine subgroups and interactions<br>(*c*) Explain how missing data were addressed<br>(*d*) If applicable, explain how loss to follow-up was addressed<br>(*e*) Describe any sensitivity analyses	5
**Results**			
Participants	13*	(a) Report numbers of individuals at each stage of study—eg numbers potentially eligible, examined for eligibility, confirmed eligible, included in the study, completing follow-up, and analysed<br>(b) Give reasons for non-participation at each stage<br>(c) Consider use of a flow diagram	4
Descriptive data	14*	(a) Give characteristics of study participants (eg demographic, clinical, social) and information on exposures and potential confounders<br>(b) Indicate number of participants with missing data for each variable of interest<br>(c) Summarise follow-up time (eg, average and total amount)	6
Outcome data	15*	Report numbers of outcome events or summary measures over time	7

For peer review only - http://bmjopen.bmj.com/site/about/guidelines.xhtml

Main results	16	(*a*) Give unadjusted estimates and, if applicable, confounder-adjusted estimates and their precision (eg, 95% confidence interval). Make clear which confounders were adjusted for and why they were included<br>(*b*) Report category boundaries when continuous variables were categorized<br>(*c*) If relevant, consider translating estimates of relative risk into absolute risk for a meaningful time period	7
Other analyses	17	Report other analyses done—eg analyses of subgroups and interactions, and sensitivity analyses	7,8
**Discussion**			
Key results	18	Summarise key results with reference to study objectives	8
Limitations	19	Discuss limitations of the study, taking into account sources of potential bias or imprecision. Discuss both direction and magnitude of any potential bias	9
Interpretation	20	Give a cautious overall interpretation of results considering objectives, limitations, multiplicity of analyses, results from similar studies, and other relevant evidence	8,9
Generalisability	21	Discuss the generalisability (external validity) of the study results	9
**Other information**			
Funding	22	Give the source of funding and the role of the funders for the present study and, if applicable, for the original study on which the present article is based	1

*Give information separately for exposed and unexposed groups.

Note: An Explanation and Elaboration article discusses each checklist item and gives methodological background and published examples of transparent reporting. The STROBE checklist is best used in conjunction with this article (freely available on the Web sites of PLoS Medicine at http://www.plosmedicine.org/, Annals of Internal Medicine at http://www.annals.org/, and Epidemiology at http://www.epidem.com/). Information on the STROBE Initiative is available at http://www.strobe-statement.org.

For peer review only

BMJ Open

Impact of the COVID-19 pandemic on intimate partner violence during pregnancy: evidence from a multi-methods study of recently pregnant women in Ethiopia

Journal:	*BMJ Open*
Manuscript ID	bmjopen-2021-055790.R2
Article Type:	Original research
Date Submitted by the Author:	28-Feb-2022
Complete List of Authors:	Wood, Shannon; Johns Hopkins University Bloomberg School of Public Health<br>Yirgu, Robel; Addis Ababa University<br>Wondimagegnehu, Abigiya; Addis Ababa University<br>Qian, Jiage; Johns Hopkins University Bloomberg School of Public Health<br>Milkovich, Rachel; Johns Hopkins University Bloomberg School of Public Health<br>Decker, Michele; Johns Hopkins University Bloomberg School of Public Health<br>Glass, Nancy; Johns Hopkins University School of Nursing<br>Seid, Fatuma; Ethiopia Ministry of Health<br>Zekarias, Lensa; Ethiopia Ministry of Health<br>Zimmerman, Linnea; Johns Hopkins University Bloomberg School of Public Health, Population Family and Reproductive Health
**Primary Subject Heading**:	Global health
Secondary Subject Heading:	Reproductive medicine
Keywords:	COVID-19, Public health < INFECTIOUS DISEASES, QUALITATIVE RESEARCH, PUBLIC HEALTH, REPRODUCTIVE MEDICINE, SEXUAL MEDICINE

SCHOLARONE™
Manuscripts

I, the Submitting Author has the right to grant and does grant on behalf of all authors of the Work (as defined in the below author licence), an exclusive licence and/or a non-exclusive licence for contributions from authors who are: i) UK Crown employees; ii) where BMJ has agreed a CC-BY licence shall apply, and/or iii) in accordance with the terms applicable for US Federal Government officers or employees acting as part of their official duties; on a worldwide, perpetual, irrevocable, royalty-free basis to BMJ Publishing Group Ltd ("BMJ") its licensees and where the relevant Journal is co-owned by BMJ to the co-owners of the Journal, to publish the Work in this journal and any other BMJ products and to exploit all rights, as set out in our licence.

The Submitting Author accepts and understands that any supply made under these terms is made by BMJ to the Submitting Author unless you are acting as an employee on behalf of your employer or a postgraduate student of an affiliated institution which is paying any applicable article publishing charge ("APC") for Open Access articles. Where the Submitting Author wishes to make the Work available on an Open Access basis (and intends to pay the relevant APC), the terms of reuse of such Open Access shall be governed by a Creative Commons licence – details of these licences and which Creative Commons licence will apply to this Work are set out in our licence referred to above.

Other than as permitted in any relevant BMJ Author's Self Archiving Policies, I confirm this Work has not been accepted for publication elsewhere, is not being considered for publication elsewhere and does not duplicate material already published. I confirm all authors consent to publication of this Work and authorise the granting of this licence.

For peer review only

Title: Impact of the COVID-19 pandemic on intimate partner violence during pregnancy: evidence from a multi-methods study of recently pregnant women in Ethiopia

Authorship team: Shannon N. Wood[1], Robel Yirgu[2], Abigiya Wondimagegnehu[2], Jiage Qian[1], Rachel Milkovich[1], Michele R. Decker[1,3,4], Nancy Glass[4,5], Fatuma Seid[6], Lensa Zekarias[6], Linnea A. Zimmerman[1]

Affiliations:
[1] Department of Population Family and Reproductive Health, Johns Hopkins Bloomberg School of Public Health, Baltimore, Maryland, USA.
[2] Addis Ababa University School of Public Health, Addis Ababa, Ethiopia.
[3] Center for Public Health and Human Rights, Johns Hopkins Bloomberg School of Public Health, Baltimore, Maryland, USA.
[4] Johns Hopkins School of Nursing, Baltimore, Maryland, USA.
[5] Center for Global Health, Johns Hopkins University, Baltimore, Maryland, USA.
[6] Ethiopia Federal Ministry of Health, Addis Ababa, Ethiopia.

Corresponding Author:
Shannon N. Wood
615 N. Wolfe Street
Baltimore, MD
swood@jhu.edu

Word count: 3,151
Figures: 1
Tables: 2

For peer review only

Abstract

Objectives: This multi-methods study aimed to: 1) compare the prevalence of intimate partner violence (IPV) during pregnancy pre- and during the COVID-19 pandemic using quantitative data; and 2) contextualize pregnant women's IPV experiences during the COVID-19 pandemic through supplemental interviews.

Design: Quantitative analyses utilize data from Performance Monitoring for Action (PMA)-Ethiopia, a cohort of 2,873 pregnant women that collects data at pregnancy, 6-weeks, 6-months, and 1-year postpartum. Following 6-week postpartum survey, in-depth semi-structured interviews contextualized experiences of IPV during pregnancy with a subset of participants (n=24).

Participants: All pregnant women residing within six regions of Ethiopia, covering 91% of the population, were eligible for the cohort study (n=2,868 completed baseline survey). Quantitative analyses were restricted to the 2,388 women with complete six-week survey data (retention=82.7%). A purposive sampling frame was used to select qualitative participants on baseline survey data, with inclusion criteria specifying completion of quantitative 6-week interview after the onset of the COVID-19 pandemic, and indication of IPV experience.

Interventions: A State of Emergency in Ethiopia was declared in response to the COVID-19 pandemic approximately halfway through 6-week postpartum interview, enabling a natural experiment (n=1,405 pre-COVID-19; n=983 during-COVID-19).

Primary outcome measures: IPV during pregnancy was assessed via the 10-item Revised Conflict and Tactics Scale (CTS-2).

Results: One in ten women experienced any IPV during pregnancy prior to COVID-19 (10.5%), and prevalence of IPV during pregnancy increased to 15.1% during the COVID-19 pandemic (aOR=1.51; p=0.02). Stratified by residence, odds of IPV during the pandemic increased for urban women only (aOR=2.09; p=0.03), however, IPV prevalence was higher in rural regions at both timepoints. Qualitative data reveal COVID-19-related stressors namely, loss of household income and increased time spent within the household, exacerbated IPV.

Conclusions: These multi-methods results highlight the prevalent, severe violence that pregnant Ethiopian women experience, with pandemic-related increases concentrated in urban areas. Integration of IPV response and safety planning across the continuum of care can mitigate impact.

For peer review only

Article summary

Strengths and limitations of this study

- Natural experiment based on split of pre-existing cohort to examine differences in intimate partner violence (IPV) during pregnancy pre/post COVID-19 restrictions
- Large quantitative sample inclusive of all pregnant women residing within six regions of Ethiopia, covering 91% of the population
- In-depth interviews to contextualize women's experiences with IPV during pregnancy during the COVID-19 pandemic
- Groupings may not exactly estimate pre/post exposure and no women who were truly only exposed to IPV during the COVID-19 pandemic
- Qualitative interviews only occurred within two regions and are not transferable to all pregnant women experiencing IPV during pregnancy

For peer review only

Introduction

Pandemics exacerbate gender inequities, including intimate partner violence (IPV) (1–3). Evidence from previous epidemics and regional crises indicates heightened IPV via increased economic insecurity, social isolation, and exposure to perpetrators, and more limited options for garnering support (1). COVID-19 is no exception—12 out of 15 early studies indicate increases in IPV globally (4).

The pregnancy and postpartum period are pivotal timepoints for assessing and responding to IPV, given health impacts to mother and baby, and multiple points of contact with healthcare providers. Global estimates indicate that 2-14% of women experience violence during pregnancy (5,6). Pre-existing IPV may be sustained or exacerbated, and abusive partners may limit access to antenatal and postnatal care (6). IPV during pregnancy incurs profound effects, including miscarriage, premature labor, low birthweight, and maternal depression (7–10). Timely response is thus critical for this vulnerable sub-population (5,10). Linkages to woman-centered care may decrease subsequent abuse (10).

Violence prevention and response is critical within the Ethiopian context. Ethiopia Demographic and Health Survey (DHS) data indicate that 27% of ever-married women age 15-49 have experienced IPV within the past year and 4% experienced IPV during pregnancy (11). In April 2020, Ethiopia declared a State of Emergency in response to the COVID-19 pandemic, with resultant lockdown measures including, but not limited to, physical distancing, school closures, prohibition of in-house visitation outside of family members, capacity limits and masking requirements on public transportation, and discouragement of within-country travel (12); these measures may disproportionately impact urban residents due to population density, urban planning, and health-seeking behaviors (13). Further, though essential to limit infection, such lockdown measures may exacerbate pregnant women's IPV experiences (2,3). Early evidence from Amhara, Ethiopia indicated similar levels of IPV pre- and during COVID-19 (14), while 18% of antenatal care attendees in Addis Ababa reported perceived increase in violence during pregnancy since the onset of the pandemic (15). Both studies, however, were limited in generalizability due to cross-sectional design and sampling considerations.

While global evidence indicates increases in IPV, to date, no studies aimed at understanding the impact of the COVID-19 pandemic or related response measures have examined violence during pregnancy within the Ethiopian context (4). Ethical standards advocate for continued IPV monitoring using existing study infrastructures if the research aims to understand the magnitude of IPV burden and linkages with social/economic factors; informs response efforts; and meets ethical obligations (1,16). Utilizing an existing national cohort of recently pregnant women and rigorous ethical standards, this study aimed to examine how the COVID-19 pandemic affected pregnant women's experiences with IPV through 1) comparison of IPV prevalence pre- and post-COVID-19 using quantitative data and 2) contextualization of women's experiences of IPV during the COVID-19 pandemic.

Methods

Overall Study Design

This multi-methods analysis is situated within the Performance Monitoring for Action (PMA)-Ethiopia cohort study, a collaboration between Johns Hopkins Bloomberg School of Public Health (JHSPH), Addis Ababa University (AAU), and the Ethiopian Federal Ministry of Health (FMoH). PMA-Ethiopia collects quantitative data on a cohort of 2,879 pregnant women at pregnancy (any gestation), 6-weeks, 6-months, and 1-year postpartum. Enrollment into the cohort began in October 2019. The full protocol for PMA Ethiopia is detailed elsewhere (17). The present analysis utilizes quantitative 6-week postpartum data to allow for most comprehensive measurement of violence throughout the entire pregnancy period. Following 6-week interview, in-depth interviews contextualized experiences of IPV during pregnancy, among a subset of participants (n=24); the qualitative phase was not a component of the original quantitative study design, and the quantitative data did not inform qualitative analyses, and vice versa. Institutional Review Board

For peer review only - http://bmjopen.bmj.com/site/about/guidelines.xhtml

approval was obtained at both JHSPH (IRB00013278) and AAU College of Health Sciences (077/20/SPH), and protocols were implemented in line with best practices for violence research (18).

Quantitative Participants

All pregnant women residing within six regions of Ethiopia that covers 91% of the population were eligible for the cohort study (n=2,868 completed baseline survey). Quantitative analyses were restricted to the 2,388 women with complete six-week survey data (retention=82.7%). A State of Emergency in Ethiopia was declared in response to the COVID-19 pandemic approximately halfway through fielding the 6-week postpartum interview (April 8, 2020), thus enabling a natural experiment. Specifically, 1,405 6-week interviews were conducted before the onset of the COVID-19 pandemic; the remaining 983 occurred after COVID-19 emergency lockdown procedures eased in early June and data collection was able to resume.

Quantitative Measures

Intimate partner violence, the primary outcome of interest, was measured via the 10-item Revised Conflict and Tactics Scale (19), which asks about specific violence behaviors at any time during pregnancy, per best practices for violence research (18).Three violence measures were examined: 1) any IPV, 2) any physical IPV, 3) any sexual IPV; affirmative response to any behavior was classified as IPV experience. Physical and sexual IPV were derived from the following items: 1) physical IPV: "push you, shake you, or throw something at you;" "slap you;" "twist your arm or pull your hair;" "punch you with his fist or with something that could hurt you;" "kick you, drag you, or beat you up;" "try to choke you or burn you on purpose;" "threaten or attack you with a knife, gun, or other weapon" and 2) sexual IPV: "physically force you to have sexual intercourse with him when you did not want to;" "physically force you to perform any other sexual acts you did not want to;" "used threats or pressure to make you have sex when you did not want to." Any physical and any sexual IPV were not mutually exclusive.

The primary exposure variable of interest measures pre/post exposure to COVID-19 restrictions, a binary variable, indicating whether a woman completed her 6-week postpartum survey before (pre-lockdown) or after (post-lockdown) April 8, when the State of Emergency was declared and data collection paused.

Analyses were stratified by urban/rural residence given the differences in COVID-19 lockdown measures. Sociodemographic variables explored as adjustment variables were chosen on a conceptual basis, and included region, household wealth, age, parity, and education; all were examined in categorical form.

Quantitative Analysis

Descriptive statistics examined the distribution of sociodemographic characteristics by pre/post exposure; design-based F statistics assessed whether distributions were similar. Venn diagrams classified violence experiences during each timepoint. Next, bivariate distributions of each type of violence were examined by exposure, overall and stratified by urban/rural residence. Bivariate (not presented) and multivariable logistic regression models were used to examine differences in violence experience pre/during the COVID-19 pandemic and residence; for adjusted models, correlations between covariates were examined for multi-collinearity, with only residence, age, and education retained within final models. All analyses were conducted in STATA 16, with statistical significance set at $p<0.05$, and accounting for complex-survey design.

Qualitative Participants

To contextualize the impact of the COVID-19 pandemic on women's experiences of violence during pregnancy, an explanatory qualitative phase was conducted following principles for qualitative descriptive design (20). Specifically, a purposive sample of 24 women identified in the survey data to have experienced IPV were invited to participate semi-structured qualitative interviews in the month following 6-week quantitative data collection in Oromiya and SNNP regions; regions were selected based on high IPV during

For peer review only - http://bmjopen.bmj.com/site/about/guidelines.xhtml

pregnancy from baseline survey and feasibility. At the 6-week survey, participants were consented for potential follow-up specific to partner-related items. A purposive sampling frame was used to select participants on baseline survey data, with inclusion criteria specifying: 1) completion of quantitative 6-week post-COVID-19 interview; 2) indication of IPV experience via quantitative data. These eligibility criteria generated a sampling frame of 17 IPV survivors in Oromiya and 18 IPV survivors in SNNP. From this purposive sampling frame, participants were randomly selected and data collection continued until feasible sample size was met (n=14 Oromiya; n=10 SNNP) (21).

Qualitative Data Collection

Training for the qualitative phase preceded data collection with focus on probing, ethical principles for IPV research, and research team protections. Semi-structured interview guides focused on women's experiences with IPV and IPV services. Participants were called prior to interview for scheduling considerations. All interviews lasted approximately 25-30 minutes. Four trained interviewers used a structured note-taking tool to allow probing of experiences, while permitting rapid analysis for timely results (22). Interviews were conducted in Amharic, Afan Oromo, or local languages of SNNP with the help of local translators. Immediately post-interview, interviewers typed and translated field notes.

Qualitative Analysis

Two researchers trained in qualitative analysis coded 24 structured notes using Atlas.ti software. Inductive thematic analysis was used to identify themes and sub-themes and to create an initial set of codes. Dual coding and retro-coding were used to enhance agreement between coders. Coders also met and collaborated regularly with the field research team to discuss and clarify interpretation. Coding was complete when saturation of themes was achieved (23); illustrative quotes were then downloaded from Atlas.ti and organized in matrices of code themes that were organized by IPV experience.

Patient and Public Involvement

Community members are not directly involved in PMA-Ethiopia research, however, a Project Advisory Board, including members of the Federal Ministry of Health, health providers, and key stakeholders, are consulted about country-specific priorities, including IPV, during survey development. Results are disseminated to the advisory board to inform action.

Results

Quantitative Results

Demographic characteristics of study participants by pre/post exposure are presented in Table 1. Significant differences were observed for parity only, where women of higher parity were more likely to be interviewed pre-COVID-19.

Figure 1 displays overlap of types of violence experienced by women during pregnancy, by exposure. At both timepoints, sexual IPV (7.4%$_{\text{pre-COVID-19}}$; 9.8%$_{\text{during-COVID-19}}$) was higher than physical IPV (5.0%$_{\text{pre-COVID-19}}$; 7.8%$_{\text{during-COVID-19}}$) and often occurred in isolation.

[insert Figure 1 here]

Bivariate and logistic regression results comparing pre-COVID-19 IPV to during COVID-19 IPV, overall and by residence, are presented in Table 2. One in ten women experienced any IPV during pregnancy prior to COVID019 (10.5%), and this proportion increased to 15.1% during the COVID-19 pandemic (aOR during COVID-19 compared to pre-COVID-19=1.51; 95% CI=1.06-2.15; p=0.02). When stratified by residence, odds of IPV during COVID-19, compared to pre-COVID-19, increased for urban women (aOR=2.09; 95%CI=1.10-3.96; p=0.03), while odds slightly attenuated for rural women, but the difference did not achieve statistical significance (aOR=1.43; 95% CI=0.96-2.13; p=0.08). IPV prevalence was

For peer review only - http://bmjopen.bmj.com/site/about/guidelines.xhtml

consistently higher among rural women at both time points (16.2% during COVID-19, 11.9% pre-COVID-19 for rural women vs. 10.9% during COVID-19, 5.6% pre-COVID-19 for urban women).

In examining violence sub-types, physical IPV increased 60% during COVID-19, compared to pre-COVID-19, in the overall sample (aOR=1.60; 95% CI=1.07-2.40; p=0.02); this increase was greater than two-fold in urban areas (aOR=2.46; 95% CI=1.18-5.10; p=0.02). Statistically significant changes by pre/post exposure were not observed for physical violence in rural areas or for sexual violence.

Qualitative Results

Qualitative data indicate that the COVID-19 pandemic and implementation of local lockdown measures affected IPV within relationships differentially based on husband's job status. Specifically, women whose husbands experienced loss of income or employment due to the pandemic often reported that the COVID-19 pandemic put stress on their relationship and increased instances of physical violence. These stressors were described by an 18-year-old woman in Oromiya who had just given birth to her first child:

> Since he was not working, he didn't have money; it was difficult for us to pay the house rent, it was difficult to buy food. These problems created conflict, then he insulted and hit me.

Further, many women experienced an increase in verbal abuse due to COVID-19-related stressors. Several participants shared that the main reason for conflict prior to the pandemic were disagreements about household responsibilities, suggesting that the COVID-19 pandemic exacerbated this tension. This increase in household tension due to restrictions on movement was discussed by a 36-year-old woman with four children in rural SNNP:

> The violence increased because we stayed together at our house because there was movement restriction in our village so he couldn't go to the market to sell goats and sheep. So, our income decreased a lot and because of that we had arguments most of the time.

The pandemic also changed the amount of time that spouses spent at home together, most often due to a husband's loss of employment. One 29-year-old participant from rural Oromiya disclosed that this gave her husband an opportunity to further control her activities and to engage in sexual violence at a high frequency:

> During corona time, he stays at home so he is happy with that because he can control all my day-to-day activities. No physical and psychological violence happened but sometimes there is sexual violence. It might be four times a week.

It should be noted that several participants stated that the pandemic had little or no impact on their relationships with their husbands, however, previous violence persisted throughout. An example of this endemic violence is described by a 37-year-old IPV survivor from rural Oromiya:

> We had a disagreement during this pregnancy. It was during coronavirus. But the cause was not related with corona. We disagreed due to children. The corona pandemic has nothing to do with our relationship.

In many instances, the violence described by women during pregnancy was severe—several examples of violence severity, particularly in relation to physical violence, are described below.

> When I became pregnant, the part of my body he hits was changed but not the frequency. He used to beat every part of my body but when I became pregnant, he especially didn't hit me around the belly. Because he cares for the baby. He slaps my face with my own hand. -36-year-old IPV survivor with four children from rural SNNP

For peer review only

He doesn’t care if I am pregnant or not. When he is very angry, he gets very emotional and throws at me whatever he finds, whether it is a chair or anything. I had stillbirth, the fetus died in the belly, though I had a spontaneous vaginal delivery. When he heard the baby died in my uterus, he got angry and thought I was the one who killed the baby. -18-year-old nulliparous IPV survivor from rural SNNP

When I was nine months pregnant; he hits me and throw me on the stone then I went to my father’s home. Immediately, my father’s neighbors took me to hospital. I was admitted there for 15 days. -18-year-old primiparous IPV survivor from urban Oromiya

Discussion

These multi-methods results highlight the prevalent, severe violence that women experience at a critical, and often vulnerable, period during their reproductive lives. Utilizing a natural experiment embedded in cohort of recently pregnant women in the six largest regions of Ethiopia, we found heightened IPV during the COVID-19 pandemic, compared to the pre-COVID-19 period, with greatest between-time impact concentrated in urban areas. Discrepancies by residence corroborate heightened cases and more COVID-19 preventive measures within urban areas (12). Qualitative data reveal the mechanisms through which pandemic-related stressors operate to exacerbate IPV, namely loss of household income and increased time spent within the household.

More than one in ten women experienced violence during pregnancy within Ethiopia ($10.5\%_{\text{pre-COVID-19}}$)—IPV during pregnancy reported by recently pregnant women in this study was substantially higher than 2016 DHS lifetime estimates (3.7%) (11). Consistent with the World Health Organization Multi-Country Study on Women’s Health and Domestic Violence and DHS (6,11), women reported higher levels of sexual violence than physical violence—notably, Ethiopia is one of the few contexts globally where this pattern is observed. Continuous monitoring within national surveillance systems and large-scale cohort studies can assist identifying who is at risk for IPV during most recent pregnancy. Utilizing best practices for violence-related research (16,18), e.g. timely linkage to woman-centered referrals, can bolster safety and minimize adverse outcomes.

Given distinct patterns of IPV by urban and rural residence, interventions must be tailored by locality and women’s circumstances. The impact of the COVID-19 pandemic on violence during pregnancy was concentrated primarily within urban settings. Immediate intervention and connection to IPV referral services should focus on urban settings, prone to closures and economic impact. As time and household finances were indicated as stressors, temporary separation and seeking support from family members and neighbors may be a feasible strategy to minimize impact of violence—this safety strategy has been useful for women experiencing IPV in urban informal settlements of Nairobi and other low- and middle-income contexts where leaving the relationship is not feasible or socially acceptable (24,25). Notably, however, IPV during pregnancy was highest for rural women at both timepoints ($16.2\%_{\text{rural}}$ vs. $10.9\%_{\text{urban}}$ during COVID-19). Heightened prevalence within rural settings likely speaks to cohesive, patriarchal community norms promoting violence against women (26); in order to ultimately reduce IPV, large-scale transformative community norms interventions, such as Communities Care or SASA! (27,28), will be required.

This study is not without limitations—namely, these groupings may not exactly estimate pre/post exposure and we have no women who were truly only exposed to IPV during the COVID-19 pandemic. Further, our sample size was limited for additional subgroup analysis to examine women most at risk for IPV during pregnancy by pre-COVID-19 and during-COVID-19 time points. To maximize women’s confidentiality and bolster safety in line with best practices for violence-related research (18), interviewers were instructed to not conduct either quantitative or qualitative interviews within the presence of a partner; given COVID-

For peer review only - http://bmjopen.bmj.com/site/about/guidelines.xhtml

19 lockdown measures and potential for controlling behaviors within abusive partnerships, our results may be an underestimate. Lastly, qualitative data collection occurred only within two of the six regions based on baseline prevalence of IPV during pregnancy—this purposive sampling strategy may have excluded women needing IPV support during the COVID-19 pandemic and is not transferable to all pregnant women experiencing IPV in Ethiopia.

While IPV during pregnancy increased overall during the COVID-19 pandemic, violence and related gender-based power disparities were prominent prior to the onset of the pandemic—accordingly, IPV prevention and response efforts in Ethiopia must be sustained in the post-pandemic era. We offer two concrete recommendations for maternal health providers. First, while antenatal care is a critical intervention point for identifying and providing care for women experiencing IPV during pregnancy, providers across the maternal and neonatal continuum of care must similarly provide support. Integration of IPV screening and psychological care into postnatal care is critical given links to postpartum depression (29,30). Clinic-based aids, including World Health Organization recommendations for clinical IPV identification and linkage to health and economic referrals, can be valuable in training providers to support survivors (31). Second, the use of safety decision-aids by providers across the continuum of care could assist in helping women assess their circumstances and level of danger, and create safety plans tailored to their situations. Safety planning with trained community health workers was found to increase safety preparedness in Kenya (32), and may be similarly valuable in rural areas of Ethiopia with trained Health Extension Workers. Clinic-based interventions to reduce and address IPV should not occur separately from other health services, but as part of larger community-led behavioral change programs with influential community members on targeting harmful gender norms and IPV, in order to ultimately empower women and girls. The COVID-19 pandemic has alerted the global community to pervasiveness of violence against women—continued momentum and investment is needed to mitigate its harmful impact.

For peer review only - http://bmjopen.bmj.com/site/about/guidelines.xhtml

Contributorship Statement: SNW designed the study, oversaw study details, and drafted the manuscript. RY oversaw all aspects of qualitative training and data collection. AW conducted training and data collection. JQ and RM conducted qualitative coding. MRD and NG served as ethical supervisors in the project. FS and LZ provided technical insight into Ethiopian IPV response systems. LAZ served as the Principal Investigator for the parent study and provided technical assistance. All authors participated in writing and approving the final manuscript.

Competing Interests: None declared.

Funding: Bill and Melinda Gates Foundation [INV 009466], the Johns Hopkins Specialized Center for Research Excellence in Sex Differences (U54AG062333), and the Foundation for Gender-Specific Medicine. Under the grant conditions of the Bill and Melinda Gates Foundation, a Creative Commons Attribution 4.0 Generic License has already been assigned to the Author Accepted Manuscript version that might arise from this submission.

Data Sharing Agreement: Quantitative data are available upon request from pmadata.org. Qualitative data are not available to maximize participant confidentiality.

Ethics Approval Statement: Johns Hopkins Bloomberg School of Public Health (IRB00013278) and Addis Ababa University College of Health Sciences (077/20/SPH)

For peer review only

References

1. Peterman A, Potts A, Donnell MO, Shah N, Oertelt-Prigione S, Gelder N Van. Working Paper 528: Pandemics and Violence Against Women and Children. Washington, DC;
2. Gelder N Van, Peterman A, Potts A, Donnell MO, Thompson K, Shah N, et al. COVID-19: Reducing the risk of infection might increase the risk of intimate partner violence. EClinicalMedicine. 2020;21.
3. Roesch E, Amin A, Gupta J, Garcia-Moreno C. Violence against women during covid-19 pandemic restrictions. BMJ. 2020;369(m1712):1–2.
4. Bourgault S, Peterman A, O'Donnell M. Violence Against Women and Children During COVID-19 — One Year On and 100 Papers In: A Fourth Research Round Up. 2021.
5. Devries KM, Kishor S, Johnson H, Stöckl H, Bacchus LJ, Garcia-Moreno C, et al. Intimate partner violence during pregnancy: analysis of prevalence data from 19 countries. Reprod Health Matters. 2010;18(36):158–70.
6. García-Moreno C, Jansen HA, Ellsberg M, Heise L, Watts C. WHO multi-country study on women's health and domestic violence against women. Geneva; 2005.
7. Hill A, Pallitto C, Mccleary-Sills J, Garcia-moreno C. A systematic review and meta-analysis of intimate partner violence during pregnancy and selected birth outcomes. Int J Gynecol Obstet. 2016;133:269–76.
8. Alhusen JL, Ray E, Sharps P, Bullock L. Intimate Partner Violence During Pregnancy: Maternal and Neonatal Outcomes. J Women. 2015;24(1):100–6.
9. Berhanie E, Gebregziabher D, Berihu H, Gerezgiher A, Kidane G. Intimate partner violence during pregnancy and adverse birth outcomes : a case-control study. 2019;1–9.
10. Chisholm C, Bullock L, Ferguson II JE. Intimate partner violence and pregnancy: epidemiology and impact. Am J Obstet Gynecol. 2017;(August):141–4.
11. Central Statistical Agency and ICF. Ethiopia Demographic and Health Survey. Addis Ababa, Ethiopia and Rockville, Maryland, USA; 2016.
12. Our World in Data. Policy Responses to the Coronavirus Pandemic--Statistics and Research [Internet]. [cited 2022 Jan 26]. Available from: https://ourworldindata.org/policy-responses-covid
13. Zimmerman LA, Desta S, Karp C, Yihdego M, Seme A, Shiferaw S, et al. Effect of the COVID-19 pandemic on health facility delivery in Ethiopia ; results from PMA Ethiopia's longitudinal panel. PLOS Glob Public Heal. 2021;1–14.
14. Tadesse AW, Tarekegn SM, Wagaw GB, Muluneh MD, Kassa AM. Prevalence and Associated Factors of Intimate Partner Violence Among Married Women During COVID-19 Pandemic Restrictions: A Community-Based Study. J Interpers Violence. 2020;
15. Teshome A, Gudu W, Bekele D, Asfaw M, Enyew R, Compton SD. Intimate partner violence among prenatal care attendees amidst the COVID-19 crisis: The incidence in Ethiopia. Int J Gynecol Obstet. 2021;153(1):45–50.
16. Peterman A, Bhatia A, Guedes A. Remote data collection on violence against women during COVID-19: A conversation with experts on ethics, measurement & research priorities.
17. Zimmerman L, Desta S, Yihdego M, Rogers A, Amogne A, Karp C, et al. Protocol for PMA-Ethiopia: A New Data Source for Cross-sectional and Longitudinal Data of Reproductive, Maternal, and Newborn Health. Accept Glob Heal Sci Pract. 2020;
18. World Health Organization (WHO). Ethical and safety recommendations for intervention research on violence against women. Geneva; 2016.
19. Straus M, Hamby S, Boney-McCoy S, Sugarman D. The Revised Conflict Tactics Scales (CTS2). J Fam Issues. 1996;17(3):283–316.
20. Bradshaw C, Atkinson S, Doody O. Employing a Qualitative Description Approach in Health Care Research. Glob Qual Nurs Res. 2017;4.
21. Guest G, Bunce A, Johnson L. How Many Interviews Are Enough? An Experiment with Data Saturation and Variability. Field methods. 2006;18(1):59–82.
22. Johnson BD, Dunlap E, Benoit E. Structured Qualitative Resesarch: Organizing "Mountains of

For peer review only

Words" for Data Analysis, both Qualitative and Quantitative. Subst Use Misuse. 2010;45(5):648–70.
23. Guest G, MacQueen K, Namey E. Applied thematic analysis. Thousand Oaks: Sage Publications; 2012.
24. Wood SN, Kennedy RS, Hameeduddin Z, Asira B, Tallam C, Akumu I, et al. "Being married doesn't mean you have to reach the end of the world:" Safety planning with intimate partner violence survivors and service providers in three urban informal settlements in Nairobi, Kenya. J Interpers Violence. 2019;Epub ahead of print.
25. Wood SN, Glass N, Decker MR. An integrative review of safety strategies for women experiencing intimate partner violence in low- and middle-income countries. Trauma, Violence, Abus. 2019;1–15.
26. Murphy M, Jones N, Yadete W, Baird S. Gender-norms, violence and adolescence: Exploring how gender norms are associated with experiences of childhood violence among young adolescents in Ethiopia. Glob Public Health. 2021;16(6):842–55.
27. Abramsky T, Devries K, Kiss L, Nakuti J, Kyegombe N, Starmann E, et al. Findings from the SASA ! Study : a cluster randomized controlled trial to assess the impact of a community mobilization intervention to prevent violence against women and reduce HIV risk in Kampala , Uganda. 2014;1–17.
28. Glass N, Perrin N, Clough A, Desgroppes A, Kaburu FN, Melton J, et al. Evaluating the communities care program: Best practice for rigorous research to evaluate gender based violence prevention and response programs in humanitarian settings. Confl Health. 2018;12(5):1–10.
29. Ashenafi W, Mengistie B, Egata G, Berhane Y. The role of intimate partner violence victimization during pregnancy on maternal postpartum depression in Eastern Ethiopia. SAGE Open Med. 2021;9:1–12.
30. Bitew T, Hanlon C, Medhin G, Fekadu A. Antenatal predictors of incident and persistent postnatal depressive symptoms in rural Ethiopia: A population-based prospective study. Reprod Health. 2019;16(1):1–9.
31. World Health Organization (WHO). Caring for women subjected to violence: a WHO curriculum for training health-care providers. Geneva; 2019.
32. Decker MR, Wood SN, Hameeduddin Z, Kennedy SR, Perrin N, Tallam C, et al. Safety decision-making and planning mobile app for intimate partner violence prevention and response: randomised controlled trial in Kenya. BMJ Glob Heal. 2020;5(002091).

For peer review only

For peer review only - http://bmjopen.bmj.com/site/about/guidelines.xhtml

Table 1. Characteristics of 6-Week Interview Participants by Exposure (n=2,388)

Demographic Characteristics	Pre-COVID-19 6-Week Interview (n=1,405)	During-COVID-19 6-Week Interview (n=983)	p-value
	n (row %)		
Region			0.28
Tigray	107 (61.5)	66 (38.5)	
Afar	23 (50.0)	23 (50.0)	
Amhara	305 (61.4)	191 (38.6)	
Oromiya	573 (55.6)	459 (44.4)	
SNNP	332 (59.6)	225 (40.4)	
Addis Ababa	45 (54.4)	38 (45.6)	
Residence			0.14
Urban	320 (61.0)	205 (39.0)	
Rural	1064 (57.2)	797 (42.8)	
Household wealth			0.23
Lower	531 (56.2)	413 (43.8)	
Higher	854 (58.2)	589 (40.8)	
Age			0.86
15-19	147 (56.5)	114 (43.6)	
20-29	736 (57.9)	534 (42.1)	
30-49	502 (58.6)	355 (41.4)	
Parity			0.001
Nulliparous	243 (50.3)	238 (49.5)	
1-2	538 (62.3)	326 (37.7)	
3+	604 (57.9)	438 (42.1)	
Education			0.95
Never attended	579 (57.8)	422 (42.2)	
Primary	554 (58.5)	393 (41.5)	
Secondary or higher	252 (57.4)	187 (42.6)	

For peer review only

For peer review only - http://bmjopen.bmj.com/site/about/guidelines.xhtml

Table 2. Bivariate and Logistic Regression Examining Type of Violence Post-COVID-19, Compared to Pre-COVID-19, Overall and by Residence

Type of Violence	Overall (n=2,388)			Urban (n=884)			Rural (n=1,504)		
	Pre (n=1,405)	During (n=983)	aOR† (95% CI)	Pre (n=540)	During (n=344)	aOR¥ (95% CI)	Pre (n=865)	During (n=639)	aOR¥ (95% CI)
	n (%)			n (%)			n (%)		
Any IPV	147 (10.5)	149 (15.1)	1.51* (1.06, 2.15)	30 (5.6)	37 (10.9)	2.09* (1.10, 3.96)	103 (11.9)	103 (16.2)	1.43 ± (0.96, 2.13)
Physical Violence	70 (5.0)	76 (7.8)	1.60* (1.07, 2.40)	19 (3.5)	27 (7.9)	2.46 * (1.18, 5.10)	47 (5.4)	49 (7.7)	1.46 (0.90, 2.36)
Sexual Violence	104 (7.4)	96 (9.8)	1.33 (0.88, 2.02)	15 (2.8)	16 (4.7)	1.70 (0.59, 4.91)	76 (8.8)	71 (11.1)	1.30 (0.83, 2.03)

†aOR adjusted for residence, age, and education; parity omitted for multicollinearity
¥aOR adjusted for age and education; parity omitted for multicollinearity
±p<0.10; *p<0.05; **p<0.01; p<0.001

For peer review only - http://bmjopen.bmj.com/site/about/guidelines.xhtml

Figure 1. Venn Diagram of Types of Violence Experienced During Pregnancy, by Pre/Post Exposure

For peer review only

For peer review only - http://bmjopen.bmj.com/site/about/guidelines.xhtml

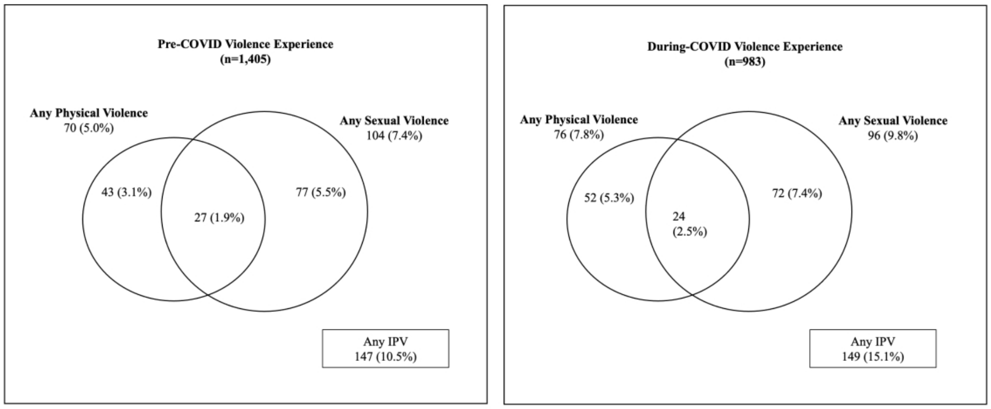

Figure 1. Venn Diagram of Types of Violence Experienced During Pregnancy, by Pre/Post Exposure

338x190mm (216 x 216 DPI)

STROBE Statement—Checklist of items that should be included in reports of ***cohort studies***

	Item No	Recommendation	Page No
**Title and abstract**	1	(*a*) Indicate the study's design with a commonly used term in the title or the abstract<br>(*b*) Provide in the abstract an informative and balanced summary of what was done and what was found	1
**1Introduction**			
Background/rationale	2	Explain the scientific background and rationale for the investigation being reported	4
Objectives	3	State specific objectives, including any prespecified hypotheses	4
**Methods**			
Study design	4	Present key elements of study design early in the paper	4
Setting	5	Describe the setting, locations, and relevant dates, including periods of recruitment, exposure, follow-up, and data collection	4
Participants	6	(*a*) Give the eligibility criteria, and the sources and methods of selection of participants. Describe methods of follow-up<br>(*b*) For matched studies, give matching criteria and number of exposed and unexposed	4
Variables	7	Clearly define all outcomes, exposures, predictors, potential confounders, and effect modifiers. Give diagnostic criteria, if applicable	5
Data sources/ measurement	8*	For each variable of interest, give sources of data and details of methods of assessment (measurement). Describe comparability of assessment methods if there is more than one group	5
Bias	9	Describe any efforts to address potential sources of bias	6
Study size	10	Explain how the study size was arrived at	4
Quantitative variables	11	Explain how quantitative variables were handled in the analyses. If applicable, describe which groupings were chosen and why	5
Statistical methods	12	(*a*) Describe all statistical methods, including those used to control for confounding<br>(*b*) Describe any methods used to examine subgroups and interactions<br>(*c*) Explain how missing data were addressed<br>(*d*) If applicable, explain how loss to follow-up was addressed<br>(*e*) Describe any sensitivity analyses	5
**Results**			
Participants	13*	(a) Report numbers of individuals at each stage of study—eg numbers potentially eligible, examined for eligibility, confirmed eligible, included in the study, completing follow-up, and analysed<br>(b) Give reasons for non-participation at each stage<br>(c) Consider use of a flow diagram	4
Descriptive data	14*	(a) Give characteristics of study participants (eg demographic, clinical, social) and information on exposures and potential confounders<br>(b) Indicate number of participants with missing data for each variable of interest<br>(c) Summarise follow-up time (eg, average and total amount)	6
Outcome data	15*	Report numbers of outcome events or summary measures over time	7

For peer review only

Main results	16	(*a*) Give unadjusted estimates and, if applicable, confounder-adjusted estimates and their precision (eg, 95% confidence interval). Make clear which confounders were adjusted for and why they were included<br>(*b*) Report category boundaries when continuous variables were categorized<br>(*c*) If relevant, consider translating estimates of relative risk into absolute risk for a meaningful time period	7
Other analyses	17	Report other analyses done—eg analyses of subgroups and interactions, and sensitivity analyses	7,8
**Discussion**			
Key results	18	Summarise key results with reference to study objectives	8
Limitations	19	Discuss limitations of the study, taking into account sources of potential bias or imprecision. Discuss both direction and magnitude of any potential bias	9
Interpretation	20	Give a cautious overall interpretation of results considering objectives, limitations, multiplicity of analyses, results from similar studies, and other relevant evidence	8,9
Generalisability	21	Discuss the generalisability (external validity) of the study results	9
**Other information**			
Funding	22	Give the source of funding and the role of the funders for the present study and, if applicable, for the original study on which the present article is based	1

*Give information separately for exposed and unexposed groups.

Note: An Explanation and Elaboration article discusses each checklist item and gives methodological background and published examples of transparent reporting. The STROBE checklist is best used in conjunction with this article (freely available on the Web sites of PLoS Medicine at http://www.plosmedicine.org/, Annals of Internal Medicine at http://www.annals.org/, and Epidemiology at http://www.epidem.com/). Information on the STROBE Initiative is available at http://www.strobe-statement.org.

For peer review only
